# Cerebrospinal Fluid as a Window into Neuroimmune Responses During Brain Infection

**DOI:** 10.3390/cells15181675

**Published:** 2026-09-16

**Authors:** Gabriela Singh, Ursula K. Rohlwink

**Affiliations:** Division of Neurosurgery, Department of Surgery, Neuroscience Institute, University of Cape Town, Observatory, Cape Town 7925, South Africa; uk.rohlwink@uct.ac.za

**Keywords:** cerebrospinal fluid, neuroinflammation, microglia, astrocytes, CNS border-associated immune cells, brain infections, tuberculous meningitis

## Abstract

Understanding neuroimmune responses during brain infections remains challenging because the site of disease (i.e., the brain) is difficult to access, immune responses within the central nervous system (CNS) are spatially and temporally compartmentalized, and peripheral immune cells have largely served as a proxy for the neuroimmune response. Cerebrospinal fluid (CSF) occupies a unique anatomical and immunological position at the interface between the brain parenchyma, CNS border compartments, and the peripheral immune system, making it an important window through which neuroinflammatory processes can be investigated in living patients. In this review, we examine evidence from CNS infections, with particular emphasis on tuberculous meningitis, demonstrating that CSF contains cellular and transcriptional profiles that differ from those observed in peripheral blood and vary according to the anatomical sampling site. We consider how CNS-resident cells, border-associated immune populations, and recruited leukocytes contribute to the neuroimmune environment of CSF. Importantly, we distinguish evidence derived from cells directly recovered from CSF from soluble biomarkers that may reflect activity in parenchymal or border compartments, and from cellular processes inferred from transcriptomic studies. We also discuss how CSF sampling and analytical approaches influence interpretation and highlight the potential of high-dimensional profiling to resolve compartmentalized neuroimmune responses during CNS infection.

## 1. Introduction

Brain infections pose a unique clinical challenge given the various disease entities, heterogeneous and non-specific presentation, and the different central nervous system (CNS) compartments that are affected [1,2]. Microglia, astrocytes (along with other brain-resident cells), and CNS border-associated immune populations interact with recruited leukocytes to coordinate pathogen recognition and inflammatory responses. These responses are essential for pathogen control, but excessive or dysregulated inflammation can also contribute to neurological injury [3].

Although once considered an immune-privileged site, the CNS is now understood to be immunologically active, with immune surveillance occurring at specialized border regions such as the meninges, cerebrospinal fluid (CSF), and perivascular space [4]. These border regions enable controlled immune cell trafficking and antigen exchange, thereby supporting dynamic communication between the CNS and the peripheral immune system [4]. Therefore, neuroinflammation is increasingly recognized as a spatially compartmentalized process likely shaped by the anatomical compartment in which it occurs, the underlying disease context, and the contributions of brain-resident cells, CNS border-associated immune populations, and infiltrating leukocytes.

Studying the neuroimmune response during brain infection remains challenging. Brain tissue sampling is rarely feasible during life and is generally restricted to selected clinical circumstances. Peripheral blood, although readily available, provides information primarily about systemic immunity and may not capture immune processes occurring within the CNS. Positioned at the interface between the brain, blood, and CNS border regions, CSF represents a valuable compartment through which these neuroimmune processes may be interrogated in vivo. In this review, we discuss the cellular and molecular processes involved in neuroinflammation during brain infections, including microglia, astrocytes, and infiltrating leukocytes, and how the cellular landscape of neuroimmunology can be studied in CSF using the emerging technologies of single-cell RNA sequencing and flow cytometry.

## 2. Literature Search Strategy

PubMed was searched for articles published within the last five years to identify literature addressing the cellular and molecular characteristics of CSF during CNS infection. Combinations of terms including “cerebrospinal fluid”, “central nervous system infection”, “neuroinflammation”, “single-cell RNA sequencing”, and “flow cytometry” were used. Additional disease-specific searches were performed for major CNS infections, including tuberculous meningitis, bacterial meningitis, viral meningitis and encephalitis, and cryptococcal meningitis. Studies were prioritized when they directly investigated human CSF during CNS infection, particularly those characterizing cellular populations, soluble inflammatory mediators, or transcriptional profiles. Evidence from peripheral blood, brain tissue, animal models, and in vitro studies was included where it provided relevant mechanistic or contextual information but was distinguished from evidence obtained directly from CSF. Reviews and foundational studies were also included where they provided contextual, essential mechanistic or methodological context. Original research articles were prioritized when discussing disease-associated cellular and molecular findings.

## 3. Why Cerebrospinal Fluid Is Uniquely Positioned to Study Neuroimmunology

CSF is a mixture of water, ions, neurotransmitters, glucose, and low concentrations of proteins, which gives rise to its clear and colorless appearance. This fluid occupies the ventricular system, subarachnoid and perivascular spaces within the CNS [5]. Traditionally regarded as a mechanical cushion and transport medium, CSF is increasingly recognized as a biologically active compartment that contributes to CNS development, homeostasis, immune surveillance, and intercellular communication [5,6].

### 3.1. Production and Composition

The choroid plexus serves as the primary source of CSF production. The plexus consists of (1) a single layer of epithelial cells connected by tight junctions and located on the basement membrane; (2) connective tissue; and (3) fenestrated blood capillaries which are relatively leaky. The capillaries enable movement of molecules between blood and epithelial cells. CSF production is driven by active ion transport across the choroid plexus epithelium. Sodium secretion into the ventricular space, supported by Na^+^/K^+^-ATPase activity, establishes osmotic and electrochemical gradients that favor the movement of Cl^−^, HCO_3_^−^, and water into the CSF. Although the choroid plexus remains the major source of CSF production, extra-choroidal sites have also been proposed to contribute to production [5]. Rather than representing a simple ultrafiltrate of plasma, CSF possesses a distinct biochemical composition that is tightly regulated through selective transport mechanisms at CNS barriers. Under physiological conditions, CSF contains low concentrations of proteins relative to plasma, together with glucose, lipids, metabolites, extracellular vesicles, and a diverse array of growth factors and signaling molecules. This tightly regulated composition enables CSF to maintain CNS homeostasis while simultaneously facilitating communication between anatomically distinct CNS compartments [5,6].

### 3.2. CSF Circulation

There are two main types of movement that have been used to describe CSF circulation, namely convective flow and pulsatile flow. Convective flow is described as unidirectional motion from the choroid plexus in the lateral ventricles, moving through the foramen of Monro into the third ventricle, and then passing through the cerebral aqueduct into the fourth ventricle [5]. Within the fourth ventricle, CSF exits the ventricular system via the three apertures (the foramina of Luschka and Magendie) to flow through the subarachnoid spaces of the brain and spinal cord before being reabsorbed into the venous circulation through the arachnoid villi and granulations on the convexity of the brain. Hydrostatic pressure gradients created by the choroid plexus (high pressure) and the arachnoid granulations (low pressure) are thought to be the driving force behind convective CSF flow. In contrast, pulsatile flow involves bidirectional movement along the spinal cord cranially and caudally with the cardiac and respiratory cycles, while within the cranial cavity flow direction and magnitude vary between regions. These dynamics are influenced by arterial pulsations, respiration, posture, and sleep. The movement of the CSF through the cranial and spinal compartments allows exposure to signals that arise from multiple anatomical regions. The ventricles and lumbar subarachnoid spaces are therefore biologically distinct compartments linked by CSF flow dynamics and characterized by physiological rostro-caudal concentration gradients. Blood-derived proteins generally increase in concentration as CSF moves from the ventricles to the spine, whereas certain brain-derived proteins may be enriched in ventricular CSF and decrease as CSF flows caudally [7,8]. These gradients may be modified by disease-specific factors like hydrocephalus, impaired CSF circulation, spinal pathology, and neuroinflammation [7].

Furthermore, the discovery of the glymphatic system has highlighted a mechanism through which CSF exchanges with brain interstitial fluid along periarterial and perivenous pathways, facilitating the clearance of metabolic waste products and proteins from the brain [5,9]. Although aspects of glymphatic physiology remain debated, there is broad consensus that bidirectional CSF-interstitial fluid exchange represents an important mechanism through which molecules generated within the brain parenchyma can enter the CSF compartment [5,9].

### 3.3. Physiological Functions

There are several complementary physiological functions through which CSF supports the CNS. One of its primary roles is mechanical protection. Although the adult human brain has an approximate mass of 1400 g, its suspension within CSF markedly reduces its effective weight because of buoyancy, thereby limiting mechanical stress on neural tissue [10]. In this way, CSF cushions the brain and helps protect it from impact against the skull during sudden head movement or trauma. In addition to its biomechanical role, CSF is essential for maintaining metabolic homeostasis within the CNS. It supplies nutrients to neurons and glial cells, removes metabolic by-products generated by cellular activity, and distributes neurotransmitters, hormones, and other biologically active molecules throughout the CNS [10]. More specifically, the glymphatic system promotes clearance of potentially neurotoxic metabolites, including amyloid-β, and impaired glymphatic function has been suggested to contribute to Alzheimer’s disease pathogenesis [11,12].

CSF also contributes to the regulation of intracranial pressure, particularly when intracranial volume changes occur as a result of changes in the blood compartment (e.g., impaired venous drainage or hemorrhage) or a space-occupying lesion. This principle is described by the Monro–Kellie doctrine, which states that the total volume within the cranial cavity remains constant and is determined by the combined volumes of brain tissue, intracranial blood, and CSF [13]. Accordingly, changes in brain or blood volume are partly compensated for by reciprocal changes in CSF volume, helping to preserve stable intracranial pressure [10]. Under conditions of neuroinflammation, however, an accumulation of CSF, hydrocephalus, may occur when the CSF circulatory pathway is disturbed by the accumulation of inflammatory exudate within the ventricular or subarachnoid spaces. This perturbs CSF flow and reabsorption and creates a clinical imperative for CSF drainage and an opportunity for CSF sampling.

An equally important physiological function of CSF is its role in immune surveillance. Although the healthy CNS contains relatively few immune cells compared to the peripheral system, immune surveillance is continuously maintained through specialized CNS borders and interfaces with the blood and CSF, including the blood-cerebrospinal fluid barrier (BCSFB), blood-spinal cord barrier (BSCB) and blood–brain barrier (BBB), the meninges, choroid plexus and perivascular spaces [4].

### 3.4. Cellular Composition of Normal CSF

Flow cytometric and single-cell studies have provided insight into the cellular composition of normal CSF. De Graaf et al. analyzed paired CSF and blood samples from 84 individuals without neurological disease. Flow cytometric analysis demonstrated a predominance of T cells among CSF leukocytes, with CD4^+^ T cells accounting for approximately 70% of CSF T cells, of which the majority displayed a central memory phenotype [14]. Granulocytes, B cells, and natural killer cells were relatively uncommon, while small populations of dendritic cells were detectable, with higher percentages of myeloid dendritic cells compared to plasmacytoid dendritic cells [14]. Recent single-cell transcriptomic work further characterized T cell populations present in healthy CSF. Pappalardo et al. identified three clusters of CD4^+^ memory T cells, two of which expressed chemokine receptor 3 (*CXCR3*) genes, which are associated with T cell trafficking [15].

The cellular composition of healthy CSF may also vary with age. Single-cell transcriptomic profiling of CSF from cognitively normal older adults has shown that healthy aging was associated with changes in transcriptional programs within CSF monocytes, including upregulation of lipid-processing genes. Whereas in cognitively impaired individuals, lipid-processing genes in monocytes were downregulated and accompanied by altered cytokine signaling to T cells [16]. Pediatric CSF cellular composition is also age-dependent, especially during early life. Studies involving neonates and young infants show that leukocyte counts are higher soon after birth than in later infancy. Kestenbaum et al. reported that infants in the first month of life had higher CSF white blood cell counts than infants aged 29–56 days [17]. Srinivasan et al. similarly showed that term and preterm infants have distinct neonatal CSF reference ranges, with protein concentrations declining with increasing postnatal age [18]. In a flow cytometric study examining CSF lymphocytes in a large pediatric cohort which included children with non-inflammatory neurological disorders, CSF γδ T cell frequencies were highest in infancy and declined with age [19]. While this study did not provide a complete phenotypic characterization of CSF cells, it suggests that some CSF T cell subsets are developmentally regulated throughout childhood.

## 4. CNS Interfaces Linking the Brain, Blood and CSF

**Figure 1:**
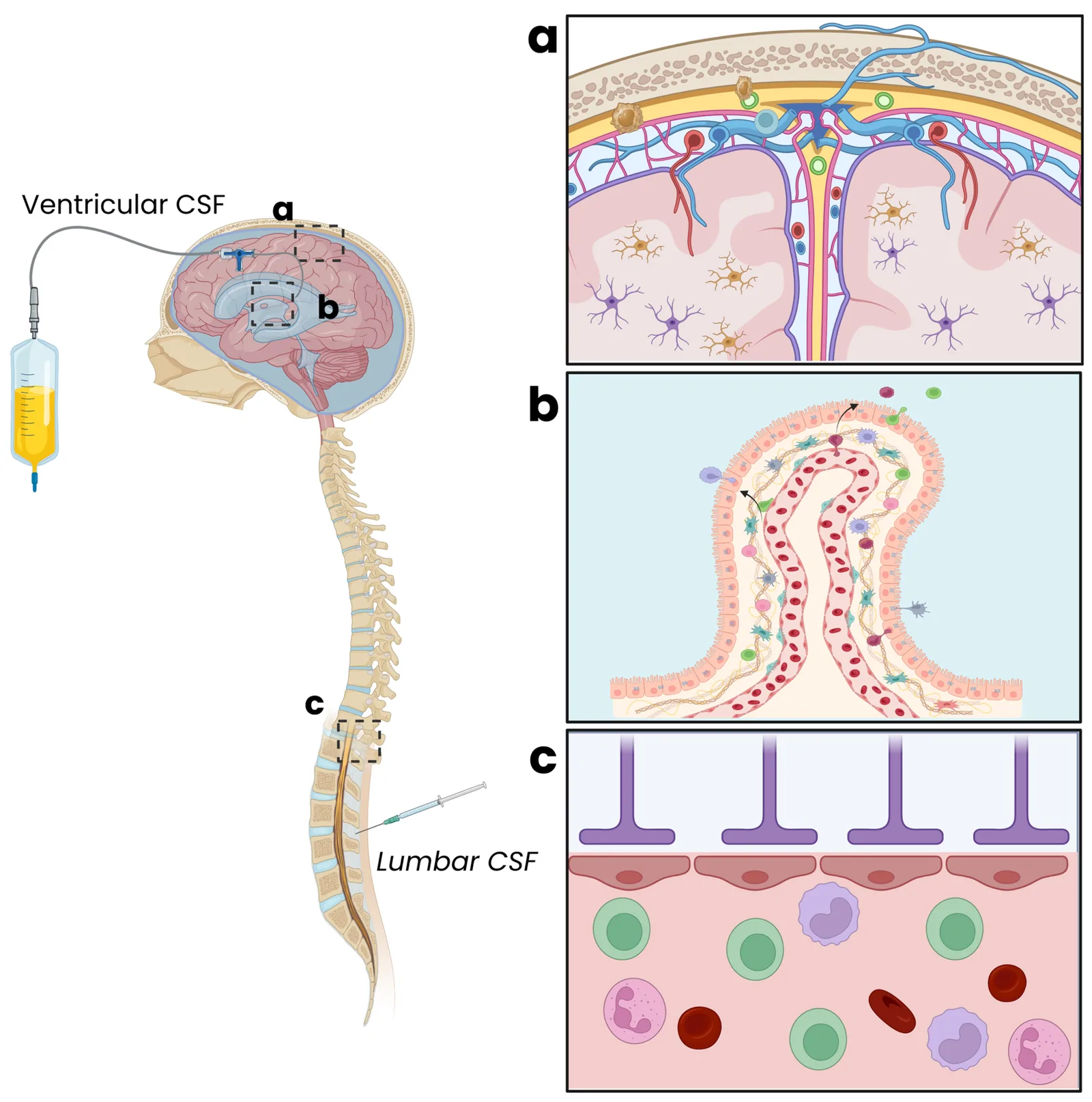
CNS interfaces linking the brain, blood, and cerebrospinal fluid. Schematic overview of the anatomical barriers and border compartments that connect the brain parenchyma, peripheral circulation, and CSF. The left panel indicates the relative positions of the barriers/interfaces, highlighting the (**a**) meninges; (**b**) blood-cerebrospinal fluid barrier at the choroid plexus; and (**c**) blood-spinal cord barrier. The various CNS barriers regulate molecular exchange and leukocyte trafficking between blood, CNS tissue, and CSF. CSF circulates through the ventricular and subarachnoid spaces and interfaces with the choroid plexus, meninges, and perivascular spaces, allowing it to integrate soluble and cellular signals from multiple CNS compartments. These anatomical relationships highlight why CSF immune profiles may reflect contributions from CNS-resident cells, border-associated immune populations, and recruited peripheral leukocytes, rather than a single site of pathology. The panels on the right show simplified representations of the cellular architecture and junctional organization of each barrier. Cellular components and junctional features are illustrated schematically and not drawn to scale.

CSF occupies a unique anatomical position between CNS barriers, subarachnoid space, and brain interstitial fluid. Consequently, CSF can integrate signals from multiple compartments and interfaces (Figure 1).

### 4.1. Barriers of the Central Nervous System

There are several barriers that participate in CNS homeostasis and protect the brain from external factors; these include the BBB, glia limitans, BCSFB, and the BSCB (Figure 1). The BBB is composed of microvascular endothelial cells that are joined together by continuous tight junctions, creating a highly regulated environment that, during homeostasis, limits immune cell trafficking of activated T cells in CSF-filled compartments [20].

Composed of astrocytes and astrocyte end feet, the glia limitans under physiological conditions serves as a barrier between the brain parenchyma and the vascular and endothelial compartments, preventing immune cells, which scan the perivascular and subarachnoid spaces, from entering the brain parenchyma [20].

The BCSFB is formed by the tight junctions between the epithelial cells of the choroid plexus, which lines all four ventricles of the brain. Similar to the BBB, the BCSFB aids in separating the CNS from systemic circulation; however, the tight junctions expressed by the choroidal epithelial cells are considerably leakier than those present in the BBB [21]. This provides a site through which peripheral immune cells can enter the CSF [21].

The BSCB shares some morphological similarities to the BBB and carries out protective and regulatory functions for the spinal cord. In comparison to the BBB, the BSCB is more permeable due to reduced expression of tight junction proteins (like claudin-5 and occludin) in the endothelial cells [22].

### 4.2. Peripheral Leukocyte Entry

Under homeostasis, leukocyte diapedesis across cerebral venules is a regulated process that can involve localized endothelial remodeling without widespread loss of endothelial barrier integrity, highlighting the distinction between cell trafficking and nonspecific barrier leakage [23]. During inflammation, endothelial activation promotes leukocyte recruitment through the increased expression of adhesion molecules and chemokines on cerebral endothelial cells [24]. In an experimental autoimmune encephalomyelitis study, leukocyte migration involves focal remodeling of the parenchymal basement membrane, including matrix metalloprotease (MMP)-2/MMP-9-mediated cleavage of dystroglycan at sites of leukocyte infiltration [25].

The BCSFB at the choroid plexus provides another important entry route for immune cells into the CSF. Unlike the BBB, the choroid plexus vasculature is fenestrated, and the barrier function is provided mainly by tight junctions between choroid plexus epithelial cells [26]. Experimental work using a reconstituted choroid plexus epithelial barrier demonstrated that activated T lymphocytes can migrate across the BCSFB and that chemokine cues enhance this process [27]. In vivo work further showed that CCR6-dependent entry of pathogenic Th17 cells through the choroid plexus can initiate CNS inflammation [28]. More recent live-imaging and single-cell transcriptomic work has shown that neutrophils and monocytes can accumulate within the choroid plexus stroma and cross the epithelial barrier into the CSF during acute neuroinflammation [29]. Furthermore, this study also demonstrated that choroid plexus epithelial cells transiently adopt specialized inflammatory programs that support immune cell recruitment, survival, and differentiation, while also regulating tight junction permeability and subsequent barrier repair [29].

The physicochemical properties of molecules also influence their transfer across CNS barriers, although permeability cannot be reduced to molecular size alone. At the BBB and BCSFB, passive diffusion generally favors smaller, more lipophilic, and less polar molecules, whereas hydrophilic or highly charged molecules are more restricted and may require specific transport mechanisms [30]. At the BCSFB, tight junctions between choroid plexus epithelial cells restrict paracellular diffusion of hydrophilic solutes, while influx, efflux, and receptor-mediated transport systems regulate the movement of ions, nutrients, and proteins [26].

Protein transfer into CSF is particularly dependent on molecular size and the concentration gradient between blood and CSF [31]. Albumin, for example, enters CSF more readily than larger immunoglobulins. Human CSF/serum data demonstrate a non-linear relationship between albumin and immunoglobulin quotients, consistent with size-dependent discrimination at the BCSFB [32].

Consequently, molecules that are poorly permeable or rapidly removed from CSF may remain at very low concentrations, whereas molecules that enter continuously or are cleared slowly can accumulate along the CSF circulation. CSF products therefore reflect the combined effects of barrier permeability, molecular properties, active transport and CSF flow and drainage.

### 4.3. CNS Border-Associated Immunity

Rather than serving as passive barriers, the meninges, perivascular spaces and the choroid plexus constitute active immunological interfaces that continuously monitor and regulate communication between the CNS and peripheral immune system. Border-associated macrophages (BAMs) represent a heterogeneous population of tissue-resident macrophages located within the meninges, perivascular spaces, and choroid plexus [33]. Recent single-cell sequencing studies have revealed distinct transcriptional profiles, developmental origins, and functional properties that distinguish BAMs from parenchymal microglia [34,35]. Strategically positioned at CNS interfaces, BAMs participate in antigen presentation, immune surveillance, and regulation of leukocyte trafficking. Their location enables them to respond rapidly to peripheral inflammatory signals while simultaneously influencing local CNS immune responses. Consequently, BAMs are increasingly recognized as critical regulators of neuroimmune communication [33].

Innate lymphoid cells (ILCs) may also contribute to CNS immunity at border zones like the meninges and choroid plexus. These cells lack clonally restricted T-cell antigen receptors and respond to microenvironment signals released from other immune cells, but are considered molecular and functional analogs of T cells [36]. Natural killer cells (NKs), ILC1s, and ILC2s appear to be enriched in the meninges, and ILC2s accumulate in the choroid plexus with aging [36]. Experimental studies suggest that these cells may limit neuroinflammation and provide a protective role after CNS injury [37,38]. Together with innate-like T cells like γδT cells, NKT cells, and mucosal-associated invariant T cells, ILCs residing at the meninges and choroid plexus appear to regulate barrier function and contribute to brain homeostasis [36].

The meninges are composed of three membranous layers that envelop the brain and spinal cord, providing an additional layer of protection to the CNS tissue and serving as an immunologically active interface between the CNS and the periphery [4,39].

The arachnoid and pia mater, collectively known as the leptomeninges, define the subarachnoid space where CSF circulates. The pia mater, which adheres directly to the CNS parenchyma, does not form a tight diffusion barrier, thereby allowing exchange between CSF and brain interstitial fluid. This permeability is critical for maintaining fluid and waste balance and for presenting CNS antigens to immune cells [4,39]. Importantly, the leptomeninges are not just a reservoir of immune cells that remain separated from CSF. Experimental studies demonstrate dynamic exchange between the leptomeningeal compartment and CSF. Schläger et al. provided direct evidence of this process using intravital imaging in experimental autoimmune encephalomyelitis [40]. Effector T cells that entered the leptomeningeal compartment from blood vessels interacted with meningeal phagocytes and could subsequently participate in CNS tissue infiltration. The study further demonstrated that CSF flow contributed to the displacement of cells from the leptomeningeal surface [40].

The distinction between leptomeningeal and dural immunity is equally important. The dura mater (outermost layer) is the most vascularized and immunologically active meningeal layer [4,39]. The highest density of hematopoietic CD45^+^ immune cells (T cells, B cells, dendritic cells, and macrophages) resides along the dural sinuses. The anatomical proximity of venous blood flow, meningeal lymphatic vessels, and immune cells in the dural sinuses creates an ideal environment for adaptive immune activation. T cells, in particular, accumulate in this region due to the presence of adhesion molecules that promote their entry and retention. Recent imaging studies conducted on both mouse and human tissue have confirmed that these cells prefer to patrol the dural sinuses over other vascular areas of the meninges [39]. B cells, including early-stage progenitors and fully differentiated plasma cells, also reside near the dural sinuses. These meningeal B cells are also present in the human meninges, particularly along the superior sagittal sinus [4]. However, most of the dura is not directly exposed to CSF because the arachnoid barrier limits free exchange between the dural compartment and CSF-filled spaces. Merlini et al. demonstrated differences between meningeal layers in CNS autoimmunity [41]. Leptomeningeal inflammation and structural changes were prominent in experimental disease and human multiple sclerosis, whereas the dura was comparatively less affected. Their findings also showed differences in vascular permeability, T cell adhesion, and antigen presentation between the two compartments [41]. In contrast, there is also evidence to suggest that immune cells located in the dura can influence CSF-associated pathology under specific disease conditions. Using fate-mapping, advanced imaging, and multi-omics approaches, Zhao et al. demonstrated how specific macrophages from the dura mater migrate into the CSF during leptomeningeal metastasis [42]. Furthermore, the authors showed that macrophage migration occurs in a matrix metalloprotease-14-dependent manner and requires the release of phosphoprotein-1 by cancer cells. Therefore, inhibiting this pathway prevents migration of macrophages and slows cancer growth [42].

Beyond the role of producing CSF and serving as an entry point for peripheral leukocytes, the choroid plexus is also an immune niche. Resident macrophages occupy distinct anatomical locations, including the stromal compartment and the apical surface of the choroid plexus epithelium. Single-cell sequencing and lineage-tracing work has revised our understanding of these populations. Du et al. identified three biologically distinct choroid plexus macrophage populations with different developmental origins and transcriptional profiles [43]. These populations were conserved across species, including corresponding macrophage subsets identified in human choroid plexus. Furthermore, during neuroinflammation, the choroid plexus macrophages exhibited type I interferon responses and produced chemokines associated with recruitment of CD8^+^ T cells [43].

Taken together, these anatomical interfaces illustrate why the cellular and molecular composition of CSF cannot be attributed to a single CNS compartment. Leukocytes may enter CSF through the BCSFB and/or BSCB, while immune populations residing within the leptomeninges, choroid plexus, and perivascular spaces may contribute directly to the CSF immune environment. Furthermore, the relative contribution of each anatomical location is likely to vary according to disease, barrier integrity, inflammatory state, and CSF flow dynamics.

## 5. Cellular Contributors to Neuroimmune Signaling Reflected in the CSF

The anatomical complexity of the CSF compartment is mirrored by the diversity of cell populations that may be detected within it. These populations do not represent a single cellular compartment. Rather, CSF may contain circulating leukocytes recruited from the periphery, immune cells associated with CNS borders, and, in some disease contexts, cells displaying phenotypic or transcriptional features associated with brain-resident populations. In addition, cellular injury and intercellular communication can generate soluble proteins and extracellular vesicles that provide indirect evidence of activity in cells.

This distinction is particularly relevant for the interpretation of brain-resident cells. Although microglia and astrocytes are central regulators of neuroimmune responses within the parenchyma, their representation in CSF differs. Microglia-like cells have been identified directly in CSF using single-cell transcriptomics and protein-based approaches, whereas astrocytic involvement is more commonly inferred from soluble proteins. The following sections consider these populations according to the strength of evidence supporting their presence in CSF, before examining their interactions with infiltrating immune cells during CNS infection.

### 5.1. Central Nervous System-Resident Cell Populations

Efforts have been made to understand the ontogeny and role of macrophages in the CNS (reviewed in greater detail by Dermitzakis et al.) [33]. Currently, macrophages in the CNS are divided into three categories: (1) CNS-associated macrophages (CAM)/BAMs, these are macrophages found in the CNS interfaces bordering the parenchyma such as the choroid plexus, meninges, and perivascular spaces; (2) Microglia, which are found in the brain parenchyma; and (3) Microglia-like cells (MLCs) are myeloid cells which cross the CNS barrier and exhibit transcriptional and phenotypic features that are similar to microglia. However, their ontogenetic origin remains uncertain and may mimic tissue-resident microglia [44] but the contributions of other CNS-associated macrophage populations like BAMS cannot be excluded. Epigenomic profiling could aid in identifying chromatin accessibility patterns that distinguish embryonically derived from bone-derived cells [45]. Trajectory-based analyses could elucidate cellular state transitions by identifying intermediate transcriptional states and relationships between microglial populations [46], lineage-tracing approaches can aid in determining cell origin [47], and multi-modal single-cell technologies that measure RNA and protein expression could offer insight into cell identity and potential ontogeny [34]. Despite both microglia and CAMs originating from the embryonic yolk sac, they can be distinguished by expression of the transmembrane 119 (TMEM119) marker, which is exclusively found in microglia.

Microglia, the resident macrophage-like cells of the CNS, are the primary immune cells of the CNS (discussed below) but are also involved in neurogenesis and formation of neuronal networks [48]. Additionally, they also assist with neuronal migration, removal of redundant neurons, and promote vessel development. During postnatal development, microglia are involved in neurogenesis, presynaptic pruning, and neuronal spine formation; they also support the development and survival of oligodendrocyte progenitor cells [48]. Assessing microglial activity has mainly relied on morphological classification, using advanced fixed and live imaging techniques. Based on established morphological features, microglia are typically classified into four categories: (1) ramified (many fine, radially branching processes); (2) primed (thicker processes with reduced secondary branching, more polarized and proliferative); (3) reactive (short, thick processes with minimal branching); and (4) amoeboid (rounded cell body lacking branches) [49]. Each of these morphological phenotypes has traditionally been interpreted as reflecting a specific functional state of microglia. For instance, ramified microglia are generally involved in immune surveillance and synaptic pruning [50]. Reactive microglia are typically associated with overt inflammatory responses, including the release of pro-inflammatory mediators and enhanced phagocytic activity [51].

However, this framework has increasingly been recognized as an oversimplification, and microglial responses are more heterogeneous than implied by the resting-to-activated continuum. Various factors, including transcriptional, epigenetic, translational, metabolic, and local environment, contribute to the function of the microglia [52]. To exemplify, single-cell, immunohistochemistry, and protein studies in brain tissue have shown that differential microglial antigen expression, including IBA1 and TMEM119 (amongst others), may suggest varying functional states and disease responses across morphological phenotypes. IBA-1 is present in all microglial phenotypes; however, increased expression of IBA1 has been associated with ischaemic injury [53,54], decreased expression has been associated with neurodegenerative diseases [55,56], and an absence of IBA1 has been found in association with metabolic conditions like hepatic encephalitis [56,57]. TMEM119 is considered a homeostatic marker, but its role in microglial activation remains unclear [58]. Autopsy data show immunohistochemical evidence of TMEM119 expression on ramified and amoeboid IBA1^+^ CD68^+^ microglia [58], while experimental work in traumatic brain injury shows decreased protein expression in contused areas (despite increased RNA expression) [59]. It has been suggested that this may be due to impaired TMEM119 translation, retraction of TMEM119-positive microglial processes accompanying the transition to an amoeboid shape [59], redistribution of the TMEM119 protein to phagosomes, or due to cleavage of the TMEM119 protein, which may limit its detection [60]. Collectively, these findings highlight the importance of synthesizing transcriptomic and genetic data with downstream protein expression and structural investigations to holistically understand microglial function.

These and other data show that the biological function of microglia is determined by more than their morphology; these cells exhibit transcriptional, proteomic, and functional heterogeneity under conditions of health and development, spatially within the brain, temporally in response to changing environmental cues, and within and across disease states (e.g., numerous disease-associated microglia have been described) [52].

### 5.2. Astrocytes

Astrocytes were once thought of as merely supportive cells but are now recognized as a highly diverse and essential population within the CNS. Depending on the region, astrocytes comprise approximately 20–40% of all glial cells in humans [61]. Astrocytes are essential for synapse formation and refinement during development. This is achieved through the secretion of extracellular matrix proteins that instruct neurons to form excitatory synapses [62]. Beyond formation, astrocytes also eliminate superfluous synapses through phagocytic pathways [63]. Astrocytes also play key roles in neurotransmitter and ion homeostasis, as well as provide metabolic support for neurons. Finally, astrocytes are fundamental in maintaining BBB integrity and neurovascular coupling [64]. Astrocytic endfeet, enriched in aquaporin 4 channels, envelop cerebral vessels and interact with endothelial cells and pericytes to maintain barrier properties. In parallel, astrocytic calcium signaling links synaptic activity to vascular tone, modulating cerebral blood flow. In summary, astrocytes serve as a central hub connecting synaptic activity, metabolic demands, and vascular supply, but importantly they also support the neuroimmune response [64].

Whereas other immune cells can be directly recovered from CSF and phenotyped using flow cytometry or single-cell approaches, intact astrocytes are not routinely recovered as a major cell population from CSF. Evidence of astrocytic involvement in CNS disease has therefore more commonly been obtained through soluble markers including glial fibrillary acidic protein (GFAP) and S100β [65].

### 5.3. Other CNS-Resident and Interface Populations

Microglia and astrocytes are not the only CNS-associated cells that may contribute to the biological information detected in CSF. Neurons, oligodendrocytes, ependymal cells, choroid plexus epithelial cells, and endothelial cells occupy distinct anatomical compartments at the brain-CSF or brain-vascular interfaces and may contribute cellular material or molecular signals to the CSF. However, evidence for their representation in CSF varies substantially.

Beyond forming the epithelial component of the BCSFB and contributing to CSF production, choroid plexus epithelial cells are capable of releasing extracellular vesicles into the CSF. An experimental study demonstrated that systemic inflammatory stimulation increases the release of choroid plexus epithelial cell-derived extracellular vesicles containing regulatory microRNAs, which can subsequently interact with astrocytes and microglia in the brain parenchyma [66]. Furthermore, elevated CSF levels of choroid plexus epithelial cell-derived extracellular vesicles were observed in an Alzheimer’s disease model. Inhibition of the release of these extracellular vesicles was also shown to prevent cognitive decline induced by amyloid β [67]. Thus, the choroid plexus may contribute to CSF neuroimmune signaling both by regulating immune cell trafficking across the BCSFB and by actively releasing signaling molecules into the CSF compartment.

Endothelial cells may also contribute indirectly to the composition of CSF. Proteomic analysis of CSF extracellular vesicles from individuals with HIV-associated neurocognitive impairment identified endothelial-associated proteins including Von Willebrand factor (VWF), intercellular adhesion molecule (ICAM), and vascular cell adhesion molecule (VCAM)-1 [68]. These findings provide evidence that vascular endothelial activity can be represented in CSF, especially during barrier dysfunction. Additionally, this study also identified neuronal and oligodendrocyte-associated proteins in CSF extracellular vesicles [68].

Ependymal cells line the ventricular CSF compartment and have been shown to respond to neuroinflammatory stimuli [69], but direct evidence that ependymal-derived cellular or molecular signals are measurable in CSF remains limited. Pericytes may represent a further, although currently less well characterized, source of vascular-derived extracellular material.

## 6. The Cellular Neuroimmune Response to Brain Infection

**Figure 2:**
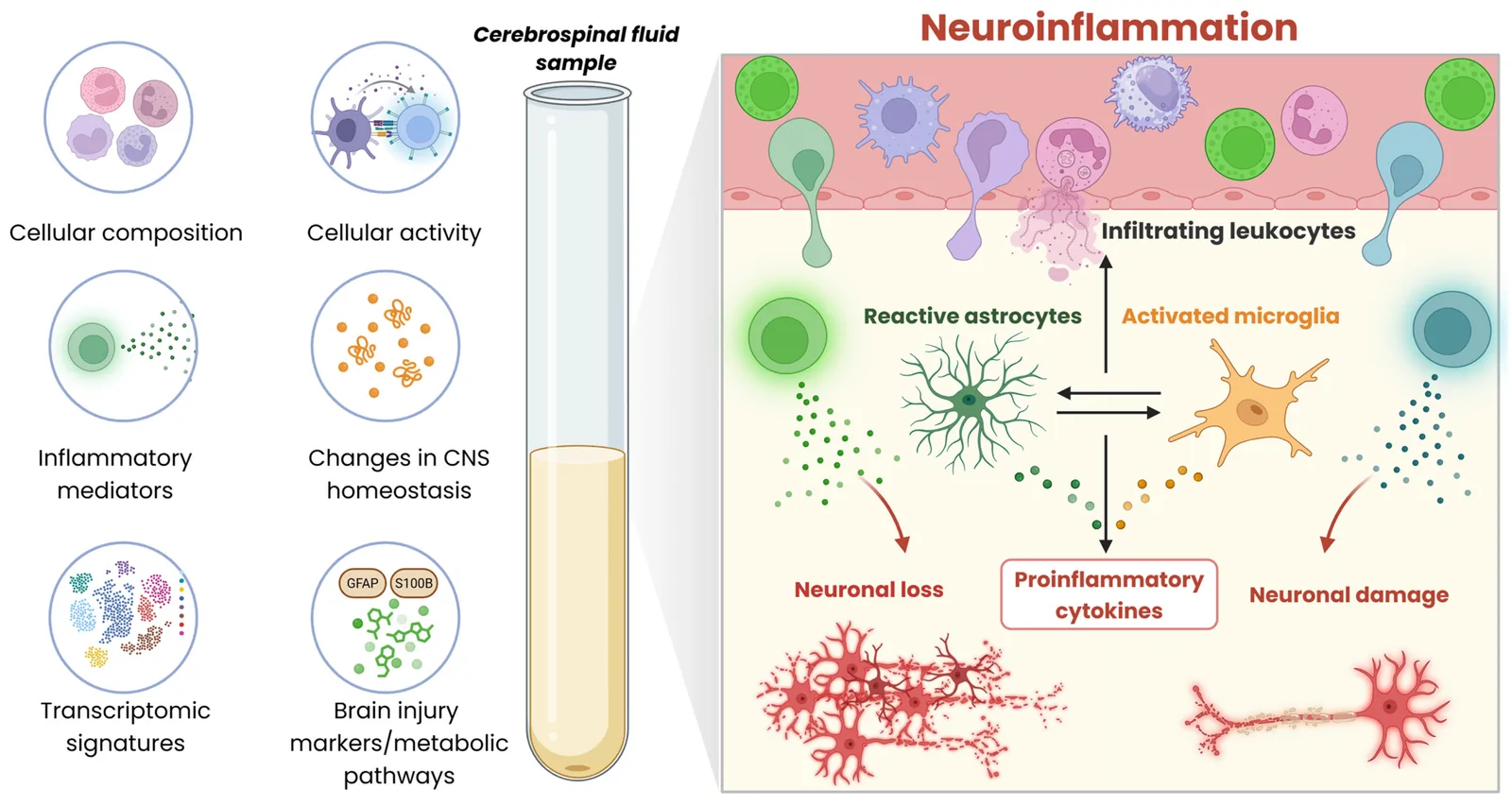
Cellular neuroimmune responses reflected in CSF during brain infection. Schematic summarizing how brain infections trigger pathogen recognition by CNS-resident and border-associated cells, cytokine and chemokine release, barrier activation, and recruitment of peripheral leukocytes into CSF. CSF immune profiles may include disease-specific shifts in neutrophils, monocytes/macrophages, T cells, natural killer cells, and microglia-like populations, together with soluble indicators of inflammation, glial activation, and neuronal injury. These signals evolve over time with pathogen burden, treatment, tissue injury, and resolution, emphasizing that CSF captures a dynamic, compartmentalized neuroimmune response rather than a static reflection of peripheral blood or brain tissue alone.

Brain infections initiate a dynamic interaction between the invading pathogen, brain-resident cells, border-associated cells, and infiltrating leukocytes. As signals through the activity of these immune cells accumulate in the CSF, this compartment provides a valuable window into the cellular/molecular drivers of neuroinflammation (Figure 2).

### 6.1. Pathogen Recognition, Barrier Responses and Leukocyte Recruitment

The initial response to CNS infection depends on recognition of pathogen-associated molecular patterns by cells at the relevant CNS interfaces and within the infected or injured tissue. These cells in turn can produce inflammatory cytokines and chemokines that modify the local vascular environment and establish gradients that direct leukocyte trafficking.

In bacterial meningitis, bacterial products and host-derived inflammatory mediators promote cerebral endothelial activation, including increased expression of adhesion molecules such as E-selectin and ICAM-1, thereby facilitating leukocyte adhesion and transmigration into the perivascular space and CSF [70,71]. Human studies have linked endothelial adhesion molecules, including E-selectin and ICAM-1, with CSF pleocytosis, protein concentrations, and intrathecal concentrations of inflammatory cytokines, supporting an association between endothelial activation and leukocyte recruitment during bacterial meningitis [72,73]. Chemokines including CXCL8 and CCL2 are also detected in CSF during bacterial meningitis and can provide chemotactic signals for recruited leukocytes [74].

Importantly, leukocyte entry does not necessarily occur through a single route. For example, experimental *Streptococcus suis* infection of a choroid plexus epithelial model induced increased expression of ICAM-1 and VCAM-1 and increased neutrophil migration [75]. Imaging further demonstrated that neutrophils preferentially used the transcellular route across the BCSFB in this model [75].

Microglia and astrocytes experimentally infected with *Mycobacterium tuberculosis* have been shown to release tumor necrosis factor (TNF)-α, interleukin (IL)-1β and IL-6, together with chemokines including CXCL10 and CCL2 that would recruit circulating leukocytes to sites of infection [76,77]. In human studies, elevated concentrations of these and other inflammatory mediators (including interferon-γ, IL-1 receptor antagonist, IL-8, IL-10, IL-12 and others) have been quantified in the CSF of patients with TBM and are considered important indicators of immune activation in the CNS [65,78]. In experimental herpes simplex virus-1 encephalitis, astrocytes and neurons have also been shown to release CXCL1, which promoted CXCR2-dependent neutrophil recruitment and was associated with increased BBB permeability, whereas CCL2-CCR2 signaling contributed to monocyte recruitment [79]. These studies demonstrate that CNS-resident cells can actively shape leukocyte recruitment and that even neurons may participate in initiating inflammatory responses.

### 6.2. A Multicellular Response Reflected in CSF

Once inflammatory signaling is established, brain-resident and infiltrating leukocytes interact through reciprocal cytokine, chemokine, and cell–cell signaling networks. The balance between cell populations and inflammatory mediators may differ according to the pathogen and the host response.

In bacterial meningitis, rapid innate immune activation and neutrophil recruitment are prominent features. Single-cell RNA sequencing has provided cellular resolution of the CSF response in bacterial meningitis. In acute bacterial meningitis, Fuchs et al. demonstrated that neutrophils acquire distinct functional features, including the induction of neutrophil T cell receptor (TCR)αβ-based V(D)J receptors in neutrophils [80]. CSF neutrophils also showed increased immune receptor repertoire diversity and phagocytic activity compared to circulating peripheral neutrophils [80]. Xiao et al. profiled the CSF of children with bacterial meningitis and identified 18 immune cell clusters, which included two neutrophil clusters, two monocyte clusters, macrophages, dendritic cells, as well as lymphocyte subsets [81]. Interestingly, this study identified FFAR2^+^TNFAIP6^+^ neutrophils and THBS1^+^IL1B^+^ monocytes, both of which were novel subtypes whose abundance correlated positively with the intensity of the inflammatory response in CSF. The cellular landscape changed over the course of disease, with myeloid populations decreasing and lymphoid populations increasing [81].

TBM demonstrates a complex interaction between innate and adaptive immune cells. Using flow cytometry in a prospective cohort of HIV-negative adults with TBM, van Laarhoven et al. demonstrated that while peripheral blood was characterized by increased neutrophils and classical monocytes, CSF leukocytes exhibited significantly higher levels of activation and were predominantly composed of αβ T cells and natural killer cells [78]. Notably, the predominance of CSF αβ T cells and natural killer cells was associated with improved survival, suggesting that CSF cellular composition may have prognostic value [78]. More recently, single-cell sequencing studies in pediatric and adult TBM patients have provided greater resolution of the CSF immune landscape. In children with TBM, Mo et al. identified a variety of immune cell clusters in paired CSF and peripheral blood and reported enrichment of a microglia-like cell population, together with Th1/Th17-associated CD4^+^ T cells, within the CSF compartment [82]. These microglia-like cells displayed evidence of complement activation and were associated with increased inflammatory mediators, including C1Q, TNF-α, and IL-6 [82]. The authors further proposed that CXCL16-CXCR6 signaling between the microglia-like cells and CD4^+^ T cells may contribute to persistent neuroinflammation in children with TBM [82]. In a similar study conducted in adult TBM patients, Tram et al. also identified immune cell clusters with differences in their distribution between compartments [83]. Highly inflammatory microglia-like cells were also found to be enriched in CSF, in addition to natural killer cells and CD8^+^ effector memory T cells. Across multiple immune cell clusters, inflammatory signaling pathways were increased, and oxidative phosphorylation pathways were reduced within CSF compared to peripheral blood [83]. These two single-cell sequencing studies provide the first evidence of microglia-like signatures in human CSF in TBM patients; however, sample sizes in both studies were small (*n* = 6 and *n* = 4, respectively), and only lumbar CSF was examined. Astrocyte characterization in TBM patients has been limited to quantification of the proteins glial fibrillary acidic protein (GFAP) and S100β in CSF. For instance, in pediatric TBM patients, raised concentrations of reactive astrocyte markers, GFAP and S100β, in CSF were associated with radiological evidence of brain injury and poor functional outcomes [65].

Compartmentalization has also been demonstrated in cryptococcal meningitis. Using multiparametric flow cytometry, Naranbhai et al. demonstrated that CSF had higher numbers of CD56^bright^ natural killer cells and non-classical monocytes and that these populations exhibited greater activation than their blood counterparts [84]. Furthermore, antifungal therapy altered peripheral immune responses more rapidly than CSF responses, highlighting persistent compartmentalized CNS inflammation during treatment [84]. More recently, Dambietz et al. also reported elevated natural killer cells and natural killer T cells in CSF of cryptococcal meningitis patients [85]. Immune abnormalities within CSF resolved only gradually during antifungal treatment [85]. Both studies highlight persistent immune activation that may continue long after initiation of treatment. Subsequent studies examining HIV-associated cryptococcal meningitis identified important relationships between CSF cellular immunity and clinical outcome. Scriven et al. showed that severe disease was associated with a relative paucity of infiltrating T cells in the CSF, whereas higher frequencies of CSF CD4^+^ and CD8^+^ T cells and increased concentrations of IL-6 were associated with lower fungal burden [86]. High intracranial pressure was similarly associated with fewer CSF T cells and greater fungal burden, suggesting that inadequate cell immunity contributes to poor disease control [86]. In a separate study in Ugandan patients with HIV-associated cryptococcal meningitis, the development of immune reconstitution inflammatory syndrome (IRIS) was associated with increasing proportions of activated CSF CD4^+^ T cells, accumulation of pro-inflammatory monocyte populations, and changes in natural killer cell activation [87].

Human CSF studies of viral brain infections have frequently focused on inflammatory mediators rather than detailed cellular phenotyping [88]. In severe viral encephalitis, CSF IL-6 and IL-8 concentrations correlate with disease severity and neurological complications [89]. Emerging transcriptomic data has, however, provided more insight into the CSF landscape. Farhadian et al., using single-cell RNA sequencing of paired CSF and blood from patients with HIV CNS infection, identified 14 immune cell clusters [90]. The majority of the clusters identified in CSF were T cells, whereas B cells, myeloid cells, and natural killer cells represented the minority of clusters. Notably, this study also identified a rare population of CSF-restricted myeloid cells with transcriptional signatures resembling neurodegenerative disease-associated microglia-like cells [90].

Rather than representing the activation of individual cell populations, neuroinflammation is an evolving process driven by coordinated communication between various cell populations from different anatomical locations.

### 6.3. Temporal Evolution of the Neuroimmune Response

A CSF sample obtained at clinical presentation represents one timepoint within an evolving interaction between pathogen replication, host immune response, treatment, tissue injury, and resolution. The temporal nature of neuroinflammation is relatively understudied in brain infections. Some longitudinal CSF studies exist in TBM [65,91], cryptococcal meningitis [92,93], and viral encephalitis [89] and offer valuable disease-specific insights into the evolution of cytokine and chemokine production over time and in response to treatment. However, serial sampling is largely uncommon in clinical studies given the invasive nature of CSF collection. Where it does occur, serial sample numbers may be limited, the timing of samples is largely dictated by clinical imperatives, and sampling may be biased to patients with more severe disease [65]. Therefore, available data are largely cross-sectional and cannot reliably distinguish transient cellular recruitment or immune activity from persistent intrathecal populations or offer conclusive evidence about the sequence or resolution of the immune response.

TBM studies provide some of the strongest evidence of the temporal evolution of CSF immune response. In a retrospective cohort of 99 patients with TBM who underwent serial lumbar punctures, CSF lymphocyte and protein concentrations changed relatively slowly following treatment, whereas polymorphonuclear cell counts and CSF glucose normalized more rapidly, demonstrating that different aspects of the CSF inflammatory profile resolve at different rates [91]. In a prospective study of adults with TBM, serial CSF-mononuclear cell ELISPOT assays demonstrated considerable variation in *M. tuberculosis*-specific IFN-γ-producing T cells [94]. Increases in CSF IFN-γ-producing T cells during the first six weeks of anti-tuberculosis treatment were observed in patients who subsequently experienced clinical deterioration, whereas patients without increasing responses did not demonstrate the same pattern [94]. In a longitudinal cytokine profiling study, Yao et al. quantified 48 cytokines in paired CSF and serum samples from patients with TBM and demonstrated that treatment was associated with significant reductions in twelve inflammatory mediators [95]. Among these, monokine induced by interferon-γ (MIG) showed negative correlations with conventional CSF measures, and along with IL-18, concentrations were significantly different from those of controls [95]. Similarly, Tomalka et al. used combined CSF cytokine and metabolomic profiling to examine immune responses at TBM diagnosis and during treatment [96]. Marked elevations in inflammatory mediator concentrations, including IL-2, TNF-α, IFN-γ and IL-1β, together with alterations in metabolic pathways involving molecules such as kynurenine, tryptophan and itaconate, were observed at diagnosis [96]. Many of the inflammatory mediators and metabolic abnormalities persisted during the two months of treatment [96]. These studies collectively suggest that immune perturbations may persist despite patients receiving treatment, suggesting delayed restoration of immune homeostasis.

Cryptococcal meningitis provides another informative example of the temporal evolution of CSF immune responses. In a prospective cytokine profiling study of HIV-uninfected cryptococcal meningitis, Jiang et al. demonstrated broad activation of Th1,2 and Th17-associated cytokine networks, with cytokine concentrations typically peaking during the first two weeks of antifungal therapy before gradually declining over time [92]. Longitudinal CSF sampling in HIV-associated cryptococcal meningitis has similarly demonstrated rapid changes in inflammatory mediators during treatment. Serial measurements during the first week showed significant decreases in VEGF, IL-6, RANTES, and IL-17 concentrations in patients treated with IFN-γ, which mirrored the decline in fungal burden [93]. More recently, Dambietz et al. used flow cytometry to analyze serial CSF samples and showed increased numbers of natural killer cells and natural killer T cells, with only a slow normalization over up to 150 days of follow-up [85].

These studies demonstrate the value of serial CSF analysis while also revealing an important limitation of the existing evidence base. Longitudinal CSF analyses have predominantly focused on conventional cell counts or soluble inflammatory mediators rather than high-dimensional profiling of defined cell populations. Consequently, relatively little is known about how individual CSF cellular states transition from initial recruitment through treatment and disease progression/resolution. This represents an important area for future investigation, particularly where serial sampling can be obtained opportunistically alongside clinically indicated lumbar puncture or ventricular drainage.

Human studies of CSF neuroimmune responses during brain infection are heterogeneous in analytical methodology, patient populations (adults versus children), and disease stage, thereby limiting direct comparison across cohorts. In Table 1, we summarize representative human studies that provide direct evidence of cellular or molecular immune responses in CSF, with emphasis on the sampling compartment, analytical approach, clinical relevance, and principal findings.

## 7. Cerebrospinal Fluid as a Distinct Neuroimmune Compartment

In this review, we use the term “compartmentalization” at several levels. Firstly, the neuroimmune response may be compartmentalized between the peripheral circulation and the CNS, resulting in different immune signatures between blood and CSF. Secondly, CSF is a distinct compartment within the CNS that integrates the signals of the brain tissue, meningitis, the choroid plexus, and perivascular spaces and therefore is not a direct surrogate for the brain. Thirdly, immune signatures may differ within the CSF compartment between ventricular and lumbar CSF because of anatomical separation, CSF flow dynamics, and disease-specific pathology.

Approaches to studying neuroinflammation have included peripheral immune profiling and tissue analysis. While post-mortem and biopsy-derived tissue studies have provided important insight into the spatial and cellular architecture of neurological diseases, these approaches are inherently limited in their ability to capture dynamic inflammatory processes over time. Post-mortem samples reflect end-stage pathology and may be influenced by tissue degradation, while ante-mortem tissue sampling is rare given that it relies on invasive procedures that typically aim to minimize tissue removal and is further limited by anatomical accessibility [98,99]. Furthermore, highly localized tissue sampling may not accurately represent broader neuroimmune interactions occurring across CNS compartments [100]. On the other hand, peripheral blood is easily accessible but demonstrates poor specificity for CNS processes because immune responses are influenced by systemic inflammation and selective trafficking across CNS barriers [20,97,101]. Data from children with TBM showed blood inflammatory mediator concentrations that were not significantly different from those of uninfected control patients, demonstrating that the cerebral immune response is compartmentalized to the brain and may not be detectable in the blood [65]. Similarly, data in adults with TBM immune reconstitution syndrome showed that cytokine and chemokine concentrations were overwhelmingly concentrated in the CSF whereas only modest corresponding concentrations were detectable in blood. This indicates that the immune response occurred locally within the CNS rather than systemically [102].

CSF occupies a unique position at the interface of brain parenchyma, CNS border regions, and the peripheral immune system. Its continuous circulation throughout the ventricular system and subarachnoid space, together with its direct exchange with brain interstitial fluid, enables CSF to integrate molecular and cellular signals arising from multiple anatomical compartments [103]. Resultantly, CSF may contain soluble mediators and cell populations derived from several anatomical sources rather than a single site of pathology, providing an opportunity to investigate the complexity of neuroinflammation at the site of disease in living patients with CNS conditions. Single-cell sequencing and immune profiling studies have provided evidence that CSF represents an important immune environment with cellular and transcriptional characteristics that differ significantly from peripheral blood. This compartmentalization of the immune response in the CNS has been demonstrated in a number of CNS conditions, including TBM, cryptococcal meningitis, and subarachnoid hemorrhage [78,84,97,104]. Paired analysis of blood and CSF samples is valuable in distinguishing passive transfer of cells and proteins across disrupted CNS barriers from selective recruitment, local proliferation, or persistence of intrathecal immune populations. Measures of BBB integrity together with immunoglobulin indices and oligoclonal band analysis can provide evidence for intrathecal antibody synthesis [7]. Similarly, the comparison of chemokine concentrations between blood and CSF may identify gradients that support immune cell trafficking into the CNS [105]. Emerging approaches like paired T cell and B cell receptor repertoire sequencing may enable assessment of clonal relationships between blood and CSF lymphocytes to help determine whether CNS populations reflect peripheral recruitment, local expansion, or persistent intrathecal immune responses [106,107]. Assessment of chemokine receptors, adhesion molecules, and trafficking molecule expression may provide additional insight into the migration and compartmentalization of immune cells within the CSF [105]. Integration of these complementary approaches with flow cytometry and single-cell transcriptomics across paired blood and CSF samples offers an increasingly sophisticated framework for evaluating neuroimmune compartmentalization. Importantly, differences between blood and CSF do not imply that the compartments are biologically independent; rather, paired sampling offers an opportunity to study the dynamics between the blood and CSF spaces while also allowing identification of brain-derived responses.

Furthermore, it is important to recognize that CSF itself is not immunologically homogeneous, as the cellular and molecular makeup is influenced not only by the generation of immune signals within the CNS but also by CSF flow dynamics. As CSF circulates through the ventricular, cranial, and spinal compartments, proteins and other biological products may accumulate according to their source, exchange pathways, and CSF flow characteristics, which may be altered under conditions of pathology and neuroinflammation [8]. CSF immune signatures will therefore reflect neuroimmune activity across multiple compartments and can vary across CSF sampling sites. Perturbations in CSF flow and reabsorption, which commonly occur in the context of neuroinflammation, may further alter the concentration and distribution of inflammatory cells and soluble mediators in the CSF. This has been observed in numerous neurological studies, which have found differences between ventricular CSF and lumbar CSF composition, indicating that distinct CSF compartments may capture different aspects of disease biology [108,109]. Transcriptomic analyses performed on paired ventricular and lumbar CSF demonstrated compartmentalization of the disease process in pediatric TBM patients, with ventricular CSF showing upregulation of pathways associated with brain injury processes like neuroexcitotoxicity, whereas lumbar CSF exhibited greater expression of pathways associated with inflammation [97]. These compartmental differences were similarly reflected at a protein level, with ventricular CSF enriched for brain injury proteins whereas lumbar CSF was characterized by approximately 4-fold higher protein concentrations and 1.6-fold higher rifampicin concentrations than ventricular CSF [65,110]. The authors hypothesize that these differences result from (1) greater permeability of the BSCB relative to the BBB, (2) slower spinal CSF flow and greater equilibration with the systemic circulation, (3) direct entry of molecules into spinal CSF, and (4) genuinely different biological processes occurring in ventricular versus lumbar compartments. Together, these findings challenge the assumption that lumbar CSF accurately reflects the brain environment in TBM [110].

This evidence suggests that ventricular and lumbar CSF should not be regarded as interchangeable specimens, and it is important to consider the sampling location when interpreting CSF immune profiles. Importantly, findings from neuroinfections, like TBM, may not generalize to all neuropathologies, as these rostro-caudal gradients are likely influenced by disease-specific factors including hydrocephalus, impaired CSF circulation, spinal pathology, and the anatomical location of the inflammation [7,8].

However, it is also important to note that research on ventricular CSF is constrained by challenges relating to sample collection, as access to the ventricular compartment requires invasive neurosurgical procedures (like the insertion of ventricular drains or shunts) which may not always be clinically indicated or available in all healthcare settings. However, centers with the opportunity to sample this compartment can offer valuable insights into brain-specific disease processes and, when paired with lumbar samples, can derive further insights into the compartmentalization of disease processes like neuroinflammation across the CNS.

## 8. Approaches for Characterizing Immune Cells in Cerebrospinal Fluid

The approaches used to investigate inflammation in the CSF have evolved considerably over the years. Early studies largely relied on routine cytological examination and measurement of total protein, glucose, and leukocyte counts [111]. Although these parameters remain essential in clinical practice, they provide limited insight into the complexity of the neuroimmune response.

CSF can be analyzed using various complementary approaches, including proteomics, metabolomics, CITE-sequencing, extracellular vesicle analysis, single-cell ATAC sequencing, imaging-based methods, and spatial or multi-omic technologies. Each of these approaches provides valuable insight into different aspects of CNS biology. However, several of these approaches, particularly single-cell ATAC sequencing, CITE-seq, spatial technologies, and integrated multi-omic analyses, remain relatively underused in CSF studies. Therefore, in this section, we will focus primarily on flow cytometry and single-cell RNA sequencing studies as these methods directly characterize the cellular immune compartment of CSF.

### 8.1. Flow Cytometry

Multiparameter flow cytometry enabled important advancements in the study of CSF immunology by enabling simultaneous phenotypic characterization of individual cell populations using combinations of fluorescently labeled antibodies. Immunophenotypic characterization, one of the most widely used applications of flow cytometry, has been applied across multiple neuroinflammatory diseases including multiple sclerosis, cryptococcal meningitis, TBM, bacterial ventriculitis, CNS autoimmune disorders, and malignancies [78,85,86,112,113,114,115]. In multiple sclerosis, flow cytometry has helped demonstrate that B-lineage cells are not only increased in the CSF but may persist longitudinally despite treatment. Eggers et al. identified clonally related CSF B cells in treatment-naïve multiple sclerosis patients, supporting the concept of local B-cell responses within the CNS [112]. Greenfield et al. further showed that a subset of CSF B cells can persist over time and resist treatment, indicating that intrathecal B-cell populations may represent a durable inflammatory reservoir rather than a transient reflection of peripheral immune activation [113].

In CNS infections, flow cytometry has provided evidence that CSF immune responses are strongly compartmentalized. In adult HIV-negative TBM patients, van Laarhoven et al. found that peripheral blood was characterized by a dominant myeloid response, with increased neutrophils and classical monocytes. In contrast, CSF leukocytes were more highly activated than blood leukocytes, and the CSF compartment showed a predominance of αβ T cells and natural killer cells, which was associated with better survival [78]. These findings illustrate that blood and CSF can reflect distinct immune processes in the same disease and that CSF cellular composition may have prognostic relevance. Similar compartmentalization has been reported in cryptococcal meningitis. Naranbhai et al. demonstrated that innate immune responses in CSF differed from those in blood during cryptococcal meningitis/HIV co-infection [84].

Flow cytometry has also been used to identify clinically relevant immune activation markers in CSF. In children with external ventricular drainage, Groselj-Grenc et al. reported that the neutrophil CD64 index in CSF could serve as a marker of bacterial ventriculitis, highlighting the potential diagnostic value of activation markers beyond routine leukocyte counts [114]. Nevertheless, CSF flow cytometry presents technical challenges including the paucicellular nature of CSF and the rapid decline in cell viability [116,117]. To address these issues, cell-stabilizing agents have been used to minimize cell loss [118,119], and cryopreservation of CSF cells has proven an effective means of preserving antigen expression and cell percentages for downstream analysis [87,120]. An important consideration when interpreting CSF immune profile data is the distinction between relative cellular composition and absolute cell concentration. Increases in the proportion of a given cell subset may not indicate true expansion of that cell type but rather depletion of other cell populations. This can be particularly relevant in paucicellular samples like CSF. Approaches that incorporate absolute quantification, including the use of counting beads, can overcome this limitation by enabling simultaneous assessment of absolute counts and cell proportions [121]. Measures that assess cellular activation and functional activity can enable a more comprehensive and biologically meaningful assessment of the cellular neuroimmune response.

Although flow cytometry therefore provides robust quantitative and phenotypic information, it remains constrained by predefined antibody panels and offers limited insight into transcriptional programs or functional cell states. Furthermore, apparent enrichment or depletion in cell populations does not necessarily indicate that these cellular profiles are driving the neuroimmune response, as flow data can reflect recruitment, retention, impaired clearance, proliferation, or altered survival of cells.

### 8.2. Single Cell RNA Sequencing

Single-cell RNA sequencing enables unbiased transcriptional profiling of thousands of individual cells simultaneously. Unlike flow cytometry, which depends upon predefined antibody panels, single-cell RNA sequencing identifies cell populations according to gene expression profiles, facilitating discovery of previously unrecognized immune cell subsets and functional states. The application of single-cell RNA sequencing to CSF has provided important evidence that CSF leukocytes are transcriptionally distinct from blood leukocytes and may include rare populations that are not readily detected using conventional phenotyping approaches. In multiple sclerosis, Schafflick et al. performed integrated single-cell analysis of paired blood and CSF leukocytes from multiple sclerosis patients and demonstrated increased transcriptional diversity present in blood, whereas increased cell-type diversity was observed in CSF, including a higher abundance of cytotoxic phenotype T helper cells [105]. Using cell set enrichment analysis, the authors also identified an increase in follicular T helper cell signatures that may support the expansion of B-lineage cells in CSF, consistent with ongoing local T- and B-cell interactions within the CNS compartment [105].

A major contribution of CSF single-cell transcriptomics has been the identification of microglia-like myeloid populations in living patients. Farhadian et al. applied single-cell RNA sequencing to paired CSF and blood samples from adults with and without HIV and identified a rare myeloid cell subset, comprising less than 5% of cells, that was found only in CSF and expressed a gene signature overlapping with neurodegenerative disease-associated microglia [90]. This finding demonstrated that CSF can contain rare CNS-associated immune populations. More recent work in prodromal Parkinson’s disease similarly reported inflamed microglia-like macrophages in the CNS, supporting the broader relevance of CSF-detectable myeloid signatures across neurodegenerative conditions [122]. The relationship between these CSF populations and bona fide brain-resident microglia remains incompletely understood as single-cell transcriptomic studies rely on transcriptomic similarity rather than direct lineage information. An alternative explanation could include the BAMS or other CNS-associated myeloid populations acquiring microglial transcriptional features in response to the specific CNS microenvironment.

Microglia-like populations have also been identified in TBM patients. Mo et al. reported highly complement-activated microglial cells associated with pediatric TBM, indicating that complement-related myeloid activation may contribute to the inflammatory and tissue-injury processes observed in this disease [82]. In adult TBM patients, Tram et al. found that although many cell subtypes were shared between peripheral blood and CSF, their relative abundance and transcriptional states differed substantially [71]. CSF was enriched for cells, CD8^+^ effector-memory T cells and CD56^bright^ natural killer cells, while the corresponding blood-enriched CD8^+^ T cell and CD56^dim^ natural killer cell populations displayed stronger cytotoxic features. Most notably, highly inflammatory microglia-like cells were restricted to the CSF compartment [83].

Despite its potential, single-cell RNA sequencing presents some ongoing challenges, including high sequencing costs, the low cellularity of CSF, sensitivity to sample handling, and the need for robust computational pipelines. Further, the data lacks the spatial resolution to localize cells or provide insight into their interactions with other cells in the native microenvironment. Caution should also be exercised when interpreting findings of rare cell populations, as their apparent biological distinctiveness could be influenced by the depth of sequencing and computational clustering methods may artifactually separate cells which form part of a broader continuum or larger population into distinct clusters. These constraints are particularly important in clinical cohorts where CSF volumes may be small and viable cells scarce. Another important limitation of CSF single-cell transcriptomic data is the potential confounding of ex vivo activation signatures stimulated by sample collection or processing. Cell isolation from tissue and sample handling may introduce rapid stress-response and immediate-early gene expression programs that can change the transcriptome relative to the native in situ phenotype [123]. Rapid sample processing and careful handling protocols can mitigate this, but this is important to consider when interpreting transcriptomic data and conducting downstream analyses [123]. Relatedly, immune cells entering the CSF from the tissue may undergo transcriptional changes when they encounter the distinct microenvironment of the CSF.

### 8.3. Acquisition, Processing and Clinical Confounders in CSF Analysis

The interpretation of CSF cell abundance and composition, transcriptomics, and protein concentration can be influenced by preanalytical variables. These include delays between sample collection and processing, transport conditions (e.g., temperature flux), centrifugation protocols, cryopreservation methods, and freeze–thaw cycles [120,124,125]. These can precipitate reduced leukocyte viability and alter representation of specific cell subsets, influence protein and RNA recovery and integrity, and influence downstream single-cell analyses [126]. Blood contamination from traumatic lumbar punctures or neurosurgery can introduce peripheral immune cells and blood-derived proteins that may confound the interpretation of intrathecal immune responses [125]. Therefore, the documentation of red blood cell counts is important in assessing sample quality and interpreting sample findings. Additionally, inflammation associated with neurosurgical procedures like the insertion of external ventricular drains and ventricular shunts could possibly contribute to the neuroimmune signal; however, if samples are taken at the start of these procedures, it is reasonable to anticipate that their contribution is unlikely to overwhelm the disease signal. Collectively, these technical considerations could influence conclusions drawn from CSF and need to be managed prospectively.

Beyond these technical considerations, there are various demographic and clinical factors that could influence CSF immune profiles. Age is an important biological confounder as the immune system changes substantially over the lifespan. In early life, innate and adaptive immune responses show developmental differences, and CNS resident cells like microglia and astrocytes may undergo age-dependent maturation and functional remodeling [46,127,128]. Similarly, aging is associated with changes in CNS immune function, including the emergence of microglial states linked to neurodegeneration. Aging microglia exhibit altered transcriptional profiles, reduced homeostatic capacity, impaired responses to injury, and increased expression of inflammatory and disease-associated pathways. Consequently, differences in CSF cellular composition, activation states, and inflammatory signatures may reflect age-related immune biology alongside disease-specific neuroinflammatory processes. Sex is also a relevant biological variable as microglia exhibit sex-dependent transcriptional, proteomic and functional differences [52]. Further research into how these differences influence susceptibility to disease is needed.

Co-morbidities like HIV co-infection may profoundly alter the systemic and intrathecal immune response. Strong evidence for this is available in the TBM literature, where HIV-associated immunosuppression has been linked to reduced CSF pleocytosis, whereas immune reconstitution following antiretroviral therapy can trigger exaggerated compartmentalized CNS inflammation characterized by marked cytokine, chemokine, and neutrophil-associated responses in the CSF [102,129]. This research also provides a strong example of the impact of treatment exposure on interpreting CSF immune signatures, where restoration of immune function led to exaggerated inflammatory responses. Corticosteroids and other immunomodulatory therapies may suppress inflammatory pathways and alter immune cell phenotypes, while effective antimicrobial treatment can modify pathogen burden and the associated host response [102,124,130]. Therefore, the timing of CSF sampling relative to symptom and treatment onset can cause variation in CSF findings.

Pathophysiological factors that influence CSF dynamics, like hydrocephalus, changes in intracranial pressure, and blood-brain barrier dysfunction, can affect the trafficking, distribution, and concentration of immune cells and soluble mediators in CSF compartments (as discussed earlier).

In summary, CSF immune signatures may not solely reflect disease-specific neuroimmunology but may also represent the combined effect of biological and clinical variables. Careful reporting and consideration of these factors is important in designing studies and interpreting CSF-based neuroimmune data.

## 9. Future Directions

Although considerable progress has been made in characterizing the CSF immune landscape, several important challenges remain. Much of the current literature is derived from lumbar CSF, whereas comparatively little is known about ventricular CSF. Direct comparisons between CNS compartments will be essential for understanding how CSF circulation and local anatomy influence neuroimmune signatures, although access to the ventricular compartment is a practical limitation to growing this line of evidence. Longitudinal CSF analyses spanning disease onset, treatment, and recovery are needed to define the temporal evolution of neuroimmune responses and distinguish protective from pathological inflammatory pathways. Such studies would be particularly informative in rapidly evolving conditions such as CNS infections. While CSF immune profiles are valuable in advancing our understanding of the neuroimmune response, their translation to clinically relevant biomarkers would require work to ascertain clear associations and thresholds for diagnosis, treatment response, and prognosis. Important work to establish the application of these profiles/biomarkers in clinical care would include standardization of sample acquisition, processing, and analysis approaches, the establishment of age- and population-specific reference values against which disease thresholds can be tested, and clinical validation and establishment of clinical utility, ideally across multi-center prospective cohorts. Important methodological considerations could include paired blood and CSF, paired lumbar and ventricular CSF, and serial sampling where clinically indicated and available, with rich metadata on patient demographics, clinical treatment, and outcome characteristics. The integration of high-dimensional flow cytometry, single-cell transcriptomics, proteomics, and spatial technologies offers a strong opportunity to establish a comprehensive picture of the neuroimmune processes reflected in the CSF. Combining these complementary approaches across diverse neurological diseases and age groups will improve our understanding of the neuroimmune response in terms of general principles, pathology-specific processes, and its evolution across the lifespan. The integration of cellular, molecular, and spatial data may help to distinguish regional and cell-specific sources of neuroimmune signals in the CSF, and enhance the value of CSF as a window into the nuances of the neuroimmune response. CSF does present some unique technical and methodological challenges which require further expertise to overcome, including invasive and limited access, limited CSF volume and cellularity, the relative scarcity of validated assay platforms, and the lack of large comparative datasets. Future work to address these limitations will require standardization of CSF sampling, storage, processing, and analytical approaches, and investment in growing well-characterized data sets.

## 10. Conclusions

Neuroinflammation due to brain infection is highly dynamic and compartmentalized and cannot be fully inferred from peripheral blood or limited available tissue analyses alone. Positioned at the interface between brain parenchyma, CNS border compartments and the peripheral immune system, CSF provides an additional and clinically accessible window into these complex immune processes. By integrating signals arising from CNS-resident cells, border-associated immune cells, and infiltrating leukocytes, CSF offers an important window through which the mechanisms that drive infection-related neuroinflammation can be inferred, while recognizing that it serves as a proxy for the brain parenchyma. As technologies continue to evolve, CSF is likely to become an increasingly important sample for studying neuroinflammation in vivo, and an important resource to identify novel therapeutic targets for modulating neuroimmune function for future clinical validation.

## Figures and Tables

**Table 1:** Comparative evidence for cellular and molecular neuroimmune responses detected in CSF during CNS infection.

Infection	CSF Sampling/Paired Blood	Disease Stage	Analytical Approach	Principal CSF Findings	Clinical Relevance	References
Tuberculous meningitis	Lumbar; very few ventricular CSF/paired blood in all studies	Acute/admission; some longitudinal sampling	Flow cytometry; scRNA-seq; inflammatory mediator analysis; metabolomic profiling	CSF-blood compartmentalization; microglia-like cells in CSF; inflammatory mediators remain elevated during treatment	CSF αβ & NK cells associated with survival; brain injury markers associated with poor outcome; host metabolism import in regulating inflammation	[78,82,96,97]
Cryptococcal Meningitis	Lumbar/paired blood in some studies	Acute/admission & longitudinal sampling	Flow cytometry; inflammatory mediator analysis	CSF-blood compartmentalization; elevated CSF NK cells; cytokine concentration changes vary between HIV-infected vs. uninfected patients on antifungal therapy	NK cells may serve as potential modulators of pathogenesis; greater fungal burden associated with fewer infiltrating T cells; combination of inflammatory mediators showed association with disease severity	[84,85,86,92]
Bacterial Meningitis	Lumbar/paired blood in some studies	Acute/admission; some longitudinal sampling	scRNA-seq;mass spectrometry	CSF neutrophils exhibit increased immune cell repertoire and phagocytic activity; novel neutrophil subtype in CSF	Abundance of novel neutrophil subtype correlated with intensity of CSF inflammatory response	[80,81]
Viral meningitis	Lumbar; paired blood in some studies	Acute/admission; longitudinal sampling	Inflammatory mediator analysis; scRNA-seq	Majority of CSF immune clusters were T cells; rare myeloid population resembling neurodegenerative disease-associated microglia-like cells	CSF IL-6 & IL-8 concentrations correlate with disease severity & neurological complications	[89,90]

The available evidence is not evenly distributed across CNS infections. TBM and cryptococcal meningitis have generated relatively detailed CSF immunological datasets, whereas cellular-resolution studies remain comparatively limited for several other CNS infections. Consequently, apparent differences in the depth of evidence across diseases should not be interpreted as differences in biological importance, but rather as differences in the extent and nature of human CSF investigation.

## Data Availability

No new data were created or analyzed in this study. Data sharing is not applicable to this article.

## Abbreviations

The following abbreviations are used in this manuscript:

BAM	Border-associated macrophage
BBB	Blood–brain barrier
BCSFB	Blood-cerebrospinal fluid barrier
BSCB	Blood-spinal cord barrier
CAM	Central nervous system-associated macrophage
CNS	Central nervous system
CSF	Cerebrospinal fluid
GFAP	Glial fibrillary acidic protein
ICAM	Intercellular adhesion molecule
IL	Interleukin
ILC	Innate lymphoid cells
IRIS	Immune reconstitution inflammatory syndrome
MLC	Microglia-like cell
MMP	Matrix metalloprotease
NK	Natural killer
NSE	Neuron-specific enolase
TBM	Tuberculous meningitis
TCR	T cell receptor
TMEM119	Transmembrane 119
TNF	Tumor necrosis factor
VCAM	Vascular cell adhesion molecule molecule
VWF	Von Willebrand factor

## References

[1] Somand D., Meurer W. (2009). Central nervous system infections. Emerg. Med. Clin. N. Am..

[2] Bowers K.M., Mudrakola V.V. (2020). Neuroinfections: Presentation, diagnosis, and treatment of meningitis and encephalitis. EMJ Neurol..

[3] Kölliker-Frers R., Udovin L., Otero-Losada M., Kobiec T., Herrera M.I., Palacios J., Razzitte G., Capani F. (2021). Neuroinflammation: An Integrating Overview of Reactive-Neuroimmune Cell Interactions in Health and Disease. Mediat. Inflamm..

[4] Croese T., Castellani G., Schwartz M. (2021). Immune cell compartmentalization for brain surveillance and protection. Nat. Immunol..

[5] Wichmann T.O., Damkier H.H., Pedersen M. (2022). A Brief Overview of the Cerebrospinal Fluid System and Its Implications for Brain and Spinal Cord Diseases. Front. Hum. Neurosci..

[6] Fame R.M., Lehtinen M.K. (2020). Emergence and Developmental Roles of the Cerebrospinal Fluid System. Dev. Cell.

[7] Reiber H. (2001). Dynamics of brain-derived proteins in cerebrospinal fluid. Clin. Chim. Acta.

[8] Reiber H. (2003). Proteins in cerebrospinal fluid and blood: Barriers, CSF flow rate and source-related dynamics. Restor. Neurol. Neurosci..

[9] Brinker T., Stopa E., Morrison J., Klinge P. (2014). A new look at cerebrospinal fluid circulation. Fluids Barriers CNS.

[10] Martin B.A., Heidari Pahlavian S., Lonser R.R., Sarntinoranont M., Bankiewicz K. (2019). Chapter 5—Anatomy and Physiology of Cerebrospinal Fluid Dynamics. Nervous System Drug Delivery.

[11] Iliff J.J., Wang M., Liao Y., Plogg B.A., Peng W., Gundersen G.A., Benveniste H., Vates G.E., Deane R., Goldman S.A. (2012). A paravascular pathway facilitates CSF flow through the brain parenchyma and the clearance of interstitial solutes, including amyloid β. Sci. Transl. Med..

[12] Hladky S.B., Barrand M.A. (2022). The glymphatic hypothesis: The theory and the evidence. Fluids Barriers CNS.

[13] Mokri B. (2001). The Monro–Kellie hypothesis. Neurology.

[14] de Graaf M.T., Smitt P.A.E.S., Luitwieler R.L., van Velzen C., van den Broek P.D.M., Kraan J., Gratama J.W. (2011). Central memory CD4+ T cells dominate the normal cerebrospinal fluid. Cytom. B Clin. Cytom..

[15] Pappalardo J.L., Zhang L., Pecsok M.K., Perlman K., Zografou C., Raddassi K., Abulaban A., Krishnaswamy S., Antel J., van Dijk D. (2020). Transcriptomic and clonal characterization of T cells in the human central nervous system. Sci. Immunol..

[16] Piehl N., van Olst L., Ramakrishnan A., Teregulova V., Simonton B., Zhang Z., Tapp E., Channappa D., Oh H., Losada P.M. (2022). Cerebrospinal fluid immune dysregulation during healthy brain aging and cognitive impairment. Cell.

[17] Kestenbaum L.A., Ebberson J., Zorc J.J., Hodinka R.L., Shah S.S. (2010). Defining Cerebrospinal Fluid White Blood Cell Count Reference Values in Neonates and Young Infants. Pediatrics.

[18] Srinivasan L., Shah S.S., Padula M.A., Abbasi S., McGowan K.L., Harris M.C. (2012). Cerebrospinal Fluid Reference Ranges in Term and Preterm Infants in the Neonatal Intensive Care Unit. J. Pediatr..

[19] Pranzatelli M.R., Allison T.J., McGee N.R., Tate E.D. (2018). Cerebrospinal fluid γδ T cell frequency is age-related: A case–control study of 435 children with inflammatory and non-inflammatory neurological disorders. Clin. Exp. Immunol..

[20] Mapunda J.A., Tibar H., Regragui W., Engelhardt B. (2022). How does the immune system enter the brain?. Front. Immunol..

[21] Thompson D., Brissette C.A., Watt J.A. (2022). The choroid plexus and its role in the pathogenesis of neurological infections. Fluids Barriers CNS.

[22] Bartanusz V., Jezova D., Alajajian B., Digicaylioglu M. (2011). The blood–spinal cord barrier: Morphology and clinical implications. Ann. Neurol..

[23] Tietz S.M., Engelhardt B. (2019). Visualizing Impairment of the Endothelial and Glial Barriers of the Neurovascular Unit during Experimental Autoimmune Encephalomyelitis In Vivo. J. Vis. Exp..

[24] Wu F., Liu L., Zhou H. (2017). Endothelial cell activation in central nervous system inflammation. J. Leukoc. Biol..

[25] Agrawal S., Anderson P., Durbeej M., van Rooijen N., Ivars F., Opdenakker G., Sorokin L.M. (2006). Dystroglycan is selectively cleaved at the parenchymal basement membrane at sites of leukocyte extravasation in experimental autoimmune encephalomyelitis. J. Exp. Med..

[26] Engelhardt B., Sorokin L. (2009). The blood-brain and the blood-cerebrospinal fluid barriers: Function and dysfunction. Semin. Immunopathol..

[27] Kivisäkk P., Mahad D.J., Callahan M.K., Trebst C., Tucky B., Wei T., Wu L., Baekkevold E.S., Lassmann H., Staugaitis S.M. (2003). Human cerebrospinal fluid central memory CD4^+^ T cells: Evidence for trafficking through choroid plexus and meninges via P-selectin. Proc. Natl. Acad. Sci. USA.

[28] Reboldi A., Coisne C., Baumjohann D., Benvenuto F., Bottinelli D., Lira S., Uccelli A., Lanzavecchia A., Engelhardt B., Sallusto F. (2009). CC chemokine receptor 6–regulated entry of TH-17 cells into the CNS through the choroid plexus is required for the initiation of EAE. Nat. Immunol..

[29] Xu H., Lotfy P., Gelb S., Pragana A., Hehnly C., Byer L.I.J., Shipley F.B., Zawadzki M.E., Cui J., Deng L. (2024). The choroid plexus synergizes with immune cells during neuroinflammation. Cell.

[30] Wu D., Chen Q., Chen X., Han F., Chen Z., Wang Y. (2023). The blood–brain barrier: Structure, regulation and drug delivery. Signal Transduct. Target. Ther..

[31] Schliep G., Felgenhauer K. (1978). Serum-CSF protein gradients, the blood-CSF barrier and the local immune response. J. Neurol..

[32] Reiber H. (1998). Cerebrospinal fluid—Physiology, analysis and interpretation of protein patterns for diagnosis of neurological diseases. Mult. Scler. J..

[33] Dermitzakis I., Theotokis P., Evangelidis P., Delilampou E., Evangelidis N., Chatzisavvidou A., Avramidou E., Manthou M.E. (2023). CNS border-associated macrophages: Ontogeny and potential implication in disease. Curr. Issues Mol. Biol..

[34] Sankowski R., Süß P., Benkendorff A., Böttcher C., Fernandez-Zapata C., Chhatbar C., Cahueau J., Monaco G., Gasull A.D., Khavaran A. (2024). Multiomic spatial landscape of innate immune cells at human central nervous system borders. Nat. Med..

[35] Van Hove H., Martens L., Scheyltjens I., De Vlaminck K., Pombo Antunes A.R., De Prijck S., Vandamme N., De Schepper S., Van Isterdael G., Scott C.L. (2019). A single-cell atlas of mouse brain macrophages reveals unique transcriptional identities shaped by ontogeny and tissue environment. Nat. Neurosci..

[36] Si Y., Zhang Y., Zuloaga K., Yang Q. (2023). The role of innate lymphocytes in regulating brain and cognitive function. Neurobiol. Dis..

[37] Fung I.T.H., Sankar P., Zhang Y., Robison L.S., Zhao X., D’Souza S.S., Salinero A.E., Wang Y., Qian J., Kuentzel M.L. (2020). Activation of group 2 innate lymphoid cells alleviates aging-associated cognitive decline. J. Exp. Med..

[38] Gadani S.P., Smirnov I., Wiltbank A.T., Overall C.C., Kipnis J. (2017). Characterization of meningeal type 2 innate lymphocytes and their response to CNS injury. J. Exp. Med..

[39] Buckley M.W., McGavern D.B. (2022). Immune dynamics in the CNS and its barriers during homeostasis and disease. Immunol. Rev..

[40] Schläger C., Körner H., Krueger M., Vidoli S., Haberl M., Mielke D., Brylla E., Issekutz T., Cabañas C., Nelson P.J. (2016). Effector T-cell trafficking between the leptomeninges and the cerebrospinal fluid. Nature.

[41] Merlini A., Haberl M., Strauß J., Hildebrand L., Genc N., Franz J., Chilov D., Alitalo K., Flügel-Koch C., Stadelmann C. (2022). Distinct roles of the meningeal layers in CNS autoimmunity. Nat. Neurosci..

[42] Zhao J., Zeng R., Li X., Lu Y., Wang Z., Peng H., Chen H., Fu M., Zhang Y., Huang Y. (2024). Dura immunity configures leptomeningeal metastasis immunosuppression for cerebrospinal fluid barrier invasion. Nat. Cancer.

[43] Du S., Nguyen K.M., Ulezko Antonova A., Fachi J.L., Rodrigues P.F., Verdiani A., Molgora M., Smirnov I., Herz J., Mamuladze T. (2026). Diversity and immune dynamics of choroid plexus macrophages are shaped by distinct developmental origins. Nat. Neurosci..

[44] Cuadros M.A., Sepulveda M.R., Martin-Oliva D., Marín-Teva J.L., Neubrand V.E. (2022). Microglia and microglia-like cells: Similar but different. Front. Cell. Neurosci..

[45] Shemer A., Grozovski J., Tay T.L., Tao J., Volaski A., Süß P., Ardura-Fabregat A., Gross-Vered M., Kim J.-S., David E. (2018). Engrafted parenchymal brain macrophages differ from microglia in transcriptome, chromatin landscape and response to challenge. Nat. Commun..

[46] Yaqubi M., Groh A.M., Dorion M.-F., Afanasiev E., Luo J.X.X., Hashemi H., Sinha S., Kieran N.W., Blain M., Cui Q.-L. (2023). Analysis of the microglia transcriptome across the human lifespan using single cell RNA sequencing. J. Neuroinflammation.

[47] Tay T.L., Mai D., Dautzenberg J., Fernández-Klett F., Lin G., Sagar N., Datta M., Drougard A., Stempfl T., Ardura-Fabregat A. (2017). A new fate mapping system reveals context-dependent random or clonal expansion of microglia. Nat. Neurosci..

[48] Prinz M., Masuda T., Wheeler M.A., Quintana F.J. (2021). Microglia and central nervous system–associated macrophages—From origin to disease modulation. Annu. Rev. Immunol..

[49] Tan Y.-L., Yuan Y., Tian L. (2020). Microglial regional heterogeneity and its role in the brain. Mol. Psychiatry.

[50] Vidal-Itriago A., Radford R.A.W., Aramideh J.A., Maurel C., Scherer N.M., Don E.K., Lee A., Chung R.S., Graeber M.B., Morsch M. (2022). Microglia morphophysiological diversity and its implications for the CNS. Front. Immunol..

[51] Reddaway J., Richardson P.E., Bevan R.J., Stoneman J., Palombo M. (2023). Microglial morphometric analysis: So many options, so little consistency. Front. Neuroinform..

[52] Paolicelli R.C., Sierra A., Stevens B., Tremblay M.-E., Aguzzi A., Ajami B., Amit I., Audinat E., Bechmann I., Bennett M. (2022). Microglia states and nomenclature: A field at its crossroads. Neuron.

[53] Michalski D., Pitsch R., Pillai D.R., Mages B., Aleithe S., Grosche J., Martens H., Schlachetzki F., Härtig W. (2017). Delayed histochemical alterations within the neurovascular unit due to transient focal cerebral ischemia and experimental treatment with neurotrophic factors. PLoS ONE.

[54] Murata Y., Sugimoto K., Yang C., Harada K., Gono R., Harada T., Miyashita Y., Higashisaka K., Katada R., Tanaka J. (2020). Activated microglia-derived macrophage-like cells exacerbate brain edema after ischemic stroke correlate with astrocytic expression of aquaporin-4 and interleukin-1 alpha release. Neurochem. Int..

[55] Swanson M.E., Scotter E.L., Smyth L.C., Murray H.C., Ryan B., Turner C., Faull R.L., Dragunow M., Curtis M.A. (2020). Identification of a dysfunctional microglial population in human Alzheimer’s disease cortex using novel single-cell histology image analysis. Acta Neuropathol. Commun..

[56] Lier J., Streit W.J., Bechmann I. (2021). Beyond Activation: Characterizing Microglial Functional Phenotypes. Cells.

[57] Dennis C.V., Sheahan P.J., Graeber M.B., Sheedy D.L., Kril J.J., Sutherland G.T. (2014). Microglial proliferation in the brain of chronic alcoholics with hepatic encephalopathy. Metab. Brain Dis..

[58] Satoh J., Kino Y., Asahina N., Takitani M., Miyoshi J., Ishida T., Saito Y. (2016). TMEM119 marks a subset of microglia in the human brain. Neuropathology.

[59] Mercurio D., Fumagalli S., Schafer M.K.-H., Pedragosa J., Ngassam L.D.C., Wilhelmi V., Winterberg S., Planas A.M., Weihe E., De Simoni M.-G. (2022). Protein Expression of the Microglial Marker Tmem119 Decreases in Association with Morphological Changes and Location in a Mouse Model of Traumatic Brain Injury. Front. Cell. Neurosci..

[60] Ruan C., Elyaman W. (2022). A New Understanding of TMEM119 as a Marker of Microglia. Front. Cell. Neurosci..

[61] Chaboub L.S., Deneen B. (2013). Astrocyte form and function in the developing central nervous system. Semin Pediatr. Neurol..

[62] Irala D., Wang S., Sakers K., Nagendren L., Severino F.P.U., Bindu D.S., Savage J.T., Eroglu C. (2024). Astrocyte-secreted neurocan controls inhibitory synapse formation and function. Neuron.

[63] Chung W.-S., Clarke L.E., Wang G.X., Stafford B.K., Sher A., Chakraborty C., Joung J., Foo L.C., Thompson A., Chen C. (2013). Astrocytes mediate synapse elimination through MEGF10 and MERTK pathways. Nature.

[64] Verkhratsky A., Butt A., Li B., Illes P., Zorec R., Semyanov A., Tang Y., Sofroniew M.V. (2023). Astrocytes in human central nervous system diseases: A frontier for new therapies. Signal Transduct. Target. Ther..

[65] Rohlwink U.K., Mauff K., Wilkinson K.A., Enslin N., Wegoye E., Wilkinson R.J., Figaji A.A. (2017). Biomarkers of cerebral injury and inflammation in pediatric tuberculous meningitis. Clin. Infect. Dis..

[66] Balusu S., Van Wonterghem E., De Rycke R., Raemdonck K., Stremersch S., Gevaert K., Brkic M., Demeestere D., Vanhooren V., Hendrix A. (2016). Identification of a novel mechanism of blood–brain communication during peripheral inflammation via choroid plexus-derived extracellular vesicles. EMBO Mol. Med..

[67] Vandendriessche C., Balusu S., Van Cauwenberghe C., Brkic M., Pauwels M., Plehiers N., Bruggeman A., Dujardin P., Van Imschoot G., Van Wonterghem E. (2021). Importance of extracellular vesicle secretion at the blood–cerebrospinal fluid interface in the pathogenesis of Alzheimer’s disease. Acta Neuropathol. Commun..

[68] Guha D., Lorenz D.R., Misra V., Chettimada S., Morgello S., Gabuzda D. (2019). Proteomic analysis of cerebrospinal fluid extracellular vesicles reveals synaptic injury, inflammation, and stress response markers in HIV patients with cognitive impairment. J. Neuroinflamm..

[69] Groh A.M.R., Caporicci-Dinucci N., Afanasiev E., Bigotte M., Lu B., Gertsvolf J., Smith M.D., Garton T., Callahan-Martin L., Allot A. (2024). Ependymal cells undergo astrocyte-like reactivity in response to neuroinflammation. J. Neurochem..

[70] Li M., Guo W., Zhu Y., Fang S., Zhang T., Shen Y. (2026). CSF Leukocytes in Intracranial Infections: Diagnostic, Mechanistic, and Therapeutic Insights for Neurocritical Care. Cell. Mol. Neurobiol..

[71] Wang S., Peng L., Gai Z., Zhang L., Jong A., Cao H., Huang S.H. (2016). Pathogenic Triad in Bacterial Meningitis: Pathogen Invasion, NF-κB Activation, and Leukocyte Transmigration that Occur at the Blood-Brain Barrier. Front. Microbiol..

[72] Fassbender K., Schminke U., Ries S., Ragoschke A., Kischka U., Fatar M., Hennerici M. (1997). Endothelial-derived adhesion molecules in bacterial meningitis: Association to cytokine release and intrathecal leukocyte-recruitment. J. Neuroimmunol..

[73] Jaber S.M., Hamed E.A., Hamed S.A. (2009). Adhesion molecule levels in serum and cerebrospinal fluid in children with bacterial meningitis and sepsis. J. Pediatr. Neurosci..

[74] Spanaus K.S., Nadal D., Pfister H.W., Seebach J., Widmer U., Frei K., Gloor S., Fontana A. (1997). C-X-C and C-C chemokines are expressed in the cerebrospinal fluid in bacterial meningitis and mediate chemotactic activity on peripheral blood-derived polymorphonuclear and mononuclear cells in vitro. J. Immunol..

[75] Wewer C., Seibt A., Wolburg H., Greune L., Schmidt M.A., Berger J., Galla H.-J., Quitsch U., Schwerk C., Schroten H. (2011). Transcellular migration of neutrophil granulocytes through the blood-cerebrospinal fluid barrier after infection with *Streptococcus suis*. J. Neuroinflamm..

[76] Rock R.B., Hu S., Gekker G., Sheng W.S., May B., Kapur V., Peterson P.K. (2005). *Mycobacterium tuberculosis*–induced cytokine and chemokine expression by human microglia and astrocytes: Effects of dexamethasone. J. Infect. Dis..

[77] Yang C.-S., Lee H.-M., Lee J.-Y., Kim J.-A., Lee S.J., Shin D.-M., Lee Y.-H., Lee D.-S., El-Benna J., Jo E.-K. (2007). Reactive oxygen species and p47phox activation are essential for the *Mycobacterium tuberculosis*-induced pro-inflammatory response in murine microglia. J. Neuroinflamm..

[78] van Laarhoven A., Dian S., van Dorp S., Purnama F., Koeken V.A., Diandini E., Utami F., Livia R., Apriani L., Ardiansyah E. (2019). Immune cell characteristics and cytokine responses in adult HIV-negative tuberculous meningitis: An observational cohort study. Sci. Rep..

[79] Michael B.D., Bricio-Moreno L., Sorensen E.W., Miyabe Y., Lian J., Solomon T., Kurt-Jones E.A., Luster A.D. (2020). Astrocyte- and Neuron-Derived CXCL1 Drives Neutrophil Transmigration and Blood-Brain Barrier Permeability in Viral Encephalitis. Cell Rep..

[80] Fuchs T., Puellmann K., Dreyfus D.H., Piehler A.P., Reuter B., Schwarzbach C., Willmann O., Yepes D., Costina V., Findeisen P. (2019). Immediate Neutrophil-Variable-T Cell Receptor Host Response in Bacterial Meningitis. Front. Neurol..

[81] Xiao H., Xiao H., Zhang Y., Guo L., Dou Z., Liu L., Zhu L., Feng W., Liu B., Hu B. (2022). High-throughput sequencing unravels the cell heterogeneity of cerebrospinal fluid in the bacterial meningitis of children. Front. Immunol..

[82] Mo S., Shi C., Cai Y., Xu M., Xu H., Xu Y., Zhang K., Zhang Y., Liu J., Che S. (2024). Single-cell transcriptome reveals highly complement activated microglia cells in association with pediatric tuberculous meningitis. Front. Immunol..

[83] Tram T.T.B., Garner L.C., Thai L.N.H., Nhat L.T.H., Thu D.D.A., Nghia H.D.T., Van L.H., Thwaites G.E., Ha V.T.N., Klenerman P. (2025). Single-cell profiling of blood and cerebrospinal fluid in tuberculous meningitis. J. Immunol..

[84] Naranbhai V., Chang C.C., Durgiah R., Omarjee S., Lim A., Moosa M.-Y.S., Elliot J.H., Ndung’u T., Lewin S.R., French M.A. (2014). Compartmentalization of innate immune responses in the central nervous system during cryptococcal meningitis/HIV coinfection. AIDS.

[85] Dambietz C., Heming M., Brix T.J., Schulte-Mecklenbeck A., Tepasse P.-R., Gross C.C., Trebicka J., Wiendl H., Meyer Zu Hörste G. (2024). Severe CSF immune cell alterations in cryptococcal meningitis gradually resolve during antifungal therapy. BMC Neurol..

[86] Scriven J.E., Graham L.M., Schutz C., Scriba T.J., Wilkinson R.J., Boulware D.R., Meintjes G., Lalloo D.G., Urban B.C. (2016). Flow Cytometry To Assess Cerebrospinal Fluid Fungal Burden in Cryptococcal Meningitis. J. Clin. Microbiol..

[87] Meya D.B., Okurut S., Zziwa G., Rolfes M.A., Kelsey M., Cose S., Joloba M., Naluyima P., Palmer B.E., Kambugu A. (2015). Cellular immune activation in cerebrospinal fluid from ugandans with cryptococcal meningitis and immune reconstitution inflammatory syndrome. J. Infect. Dis..

[88] Shen H., Liu M., Zhou H., Li Y., Guo Y., Yin Y., Zhang F., Wang J. (2024). Differential expression and significance of cytokines in cerebrospinal fluid of patients with viral encephalitis. Neuroscience.

[89] Huang P., Yang F., Dong R., Wen L., Zang Q., Song D., Guo J., Wang Y., Zhang R., Ren Z. (2025). Cerebrospinal fluid and serum cytokine profiles in severe viral encephalitis with implications for refractory status epilepticus: A retrospective observational study. Front. Immunol..

[90] Farhadian S.F., Mehta S.S., Zografou C., Robertson K., Price R.W., Pappalardo J., Chiarella J., Hafler D.A., Spudich S.S. (2018). Single-cell RNA sequencing reveals microglia-like cells in cerebrospinal fluid during virologically suppressed HIV. JCI Insight.

[91] Patel V.B., Burger I., Connolly C. (2008). Temporal evolution of cerebrospinal fluid following initiation of treatment for tuberculous meningitis. S. Afr. Med. J..

[92] Jiang Y.-K., Wang R.-Y., Zhou L.-H., Cheng J.-H., Luo Y., Zhu R.-S., Qiu W.-J., Zhao H.-Z., Wang X., Harrison T.S. (2022). Cerebrospinal fluid cytokine and chemokine patterns correlate with prognosis of HIV-uninfected cryptococcal meningitis: A prospective observational study. Front. Immunol..

[93] Jarvis J.N., Meintjes G., Bicanic T., Buffa V., Hogan L., Mo S., Tomlinson G., Kropf P., Noursadeghi M., Harrison T.S. (2015). Cerebrospinal Fluid Cytokine Profiles Predict Risk of Early Mortality and Immune Reconstitution Inflammatory Syndrome in HIV-Associated Cryptococcal Meningitis. PLoS Pathog..

[94] Park K.H., Lee M.S., Lee S.O., Choi S.H., Kim Y.S., Woo J.H., Kang J.K., Lee S.A., Kim S.H. (2014). Kinetics of T-cell-based assays on cerebrospinal fluid and peripheral blood mononuclear cells in patients with tuberculous meningitis. Korean J. Intern. Med..

[95] Yao X.-P., Hong J.-C., Jiang Z.-J., Pan Y.-Y., Liu X.-F., Wang J.-M., Fan R.-J., Yang B.-H., Zhang W.-Q., Fan Q.-C. (2024). Systemic and cerebrospinal fluid biomarkers for tuberculous meningitis identification and treatment monitoring. Microbiol. Spectr..

[96] Tomalka J., Sharma A., Smith A.G.C., Avaliani T., Gujabidze M., Bakuradze T., Sabanadze S., Jones D.P., Avaliani Z., Kipiani M. (2024). Combined cerebrospinal fluid metabolomic and cytokine profiling in tuberculosis meningitis reveals robust and prolonged changes in immunometabolic networks. Tuberculosis.

[97] Rohlwink U.K., Figaji A., Wilkinson K.A., Horswell S., Sesay A.K., Deffur A., Enslin N., Solomons R., Van Toorn R., Eley B. (2019). Tuberculous meningitis in children is characterized by compartmentalized immune responses and neural excitotoxicity. Nat. Commun..

[98] Olney K.C., Gibson K.A., Cadiz M.P., Rahimzadeh N., Swarup V., Fryer J.D. (2025). Postmortem Interval Leads to Loss of Disease-Specific Signatures in Brain Tissue. eNeuro.

[99] Ferreira P.G., Muñoz-Aguirre M., Reverter F., Cp S.G., Sousa A., Amadoz A., Sodaei R., Hidalgo M.R., Pervouchine D., Carbonell-Caballero J. (2018). The effects of death and post-mortem cold ischemia on human tissue transcriptomes. Nat. Commun..

[100] Chu D., Zhang W., Zhou X., Yin H., Yin J. (2026). The spatial architecture of neuroimmune interactions in epilepsy. Front. Immunol..

[101] Gigase F.A., Smith E., Collins B., Moore K., Snijders G.J., Katz D., Bergink V., Perez-Rodriquez M.M., De Witte L.D. (2023). The association between inflammatory markers in blood and cerebrospinal fluid: A systematic review and meta-analysis. Mol. Psychiatry.

[102] Marais S., Wilkinson K.A., Lesosky M., Coussens A.K., Deffur A., Pepper D.J., Schutz C., Ismail Z., Meintjes G., Wilkinson R.J. (2014). Neutrophil-associated central nervous system inflammation in tuberculous meningitis immune reconstitution inflammatory syndrome. Clin. Infect. Dis..

[103] Illes S. (2017). More than a drainage fluid: The role of CSF in signaling in the brain and other effects on brain tissue. Handb. Clin. Neurol..

[104] Moraes L., Grille S., Morelli P., Mila R., Trias N., Brugnini A., Lluberas N., Biestro A., Lens D. (2015). Immune cells subpopulations in cerebrospinal fluid and peripheral blood of patients with Aneurysmal Subarachnoid Hemorrhage. SpringerPlus.

[105] Schafflick D., Xu C.A., Hartlehnert M., Cole M., Schulte-Mecklenbeck A., Lautwein T., Wolbert J., Heming M., Meuth S.G., Kuhlmann T. (2020). Integrated single cell analysis of blood and cerebrospinal fluid leukocytes in multiple sclerosis. Nat. Commun..

[106] Nozuma S., Enose-Akahata Y., Johnson K.R., Monaco M.C., Ngouth N., Elkahloun A., Ohayon J., Zhu J., Jacobson S. (2021). Immunopathogenic CSF TCR repertoire signatures in virus-associated neurologic disease. JCI Insight.

[107] Ruschil C., Kemmerer C.L., Beller L., Gabernet G., Kowarik M.C. (2021). Next Generation Sequencing of Cerebrospinal Fluid B Cell Repertoires in Multiple Sclerosis and Other Neuro-Inflammatory Diseases—A Comprehensive Review. Diagnostics.

[108] Rostgaard N., Olsen M.H., Ottenheijm M., Drici L., Simonsen A.H., Plomgaard P., Gredal H., Poulsen H.H., Zetterberg H., Blennow K. (2023). Differential proteomic profile of lumbar and ventricular cerebrospinal fluid. Fluids Barriers CNS.

[109] Dong Q., Huang Z., Yu P., Song E., Chen Z., Qin F. (2022). Ventricular and lumbar cerebrospinal fluid analysis in 77 HIV-negative patients with Cryptococcal meningitis who received a ventriculoperitoneal shunt. Sci. Rep..

[110] Combrinck J., Tshavhungwe P., Rohlwink U., Enslin N., Thango N., Lazarus J., Kriegler K., Castel S., Abdelgawad N., Mcilleron H. (2023). Rifampicin and protein concentrations in paired spinal versus ventricular cerebrospinal fluid samples of children with tuberculous meningitis. J. Antimicrob. Chemother..

[111] Deisenhammer F., Bartos A., Egg R., Gilhus N., Giovannoni G., Rauer S., Sellebjerg F., Tumani H. (2011). Routine cerebrospinal fluid (CSF) analysis. European Handbook of Neurological Management.

[112] Eggers E.L., Michel B.A., Wu H., Wang S.Z., Bevan C.J., Abounasr A., Pierson N.S., Bischof A., Kazer M., Leitner E. (2017). Clonal relationships of CSF B cells in treatment-naive multiple sclerosis patients. JCI Insight.

[113] Greenfield A.L., Dandekar R., Ramesh A., Eggers E.L., Wu H., Laurent S., Harkin W., Pierson N.S., Weber M.S., Henry R.G. (2019). Longitudinally persistent cerebrospinal fluid B cells can resist treatment in multiple sclerosis. JCI Insight.

[114] Groselj-Grenc M., Derganc M., Kopitar A.N., Pavcnik M. (2019). Neutrophil CD64 index in cerebrospinal fluid as a marker of bacterial ventriculitis in children with external ventricular drainage. BMC Pediatr..

[115] Räuber S., Schulte-Mecklenbeck A., Willison A., Hagler R., Jonas M., Pul D., Masanneck L., Schroeter C.B., Golombeck K.S., Lichtenberg S. (2024). Flow cytometry identifies changes in peripheral and intrathecal lymphocyte patterns in CNS autoimmune disorders and primary CNS malignancies. J. Neuroinflamm..

[116] Subirá D., Castañón S., Aceituno E., Hernández J., Jiménez-Garófano C., Jiménez A., Jiménez A.M., Román A., Orfao A. (2002). Flow Cytometric Analysis of Cerebrospinal Fluid Samples and Its Usefulness in Routine Clinical Practice. Am. J. Clin. Pathol..

[117] de Graaf M.T., van den Broek P.D.M., Kraan J., Luitwieler R.L., van den Bent M.J., Boonstra J.G., Schmitz P.I.M., Gratama J.W., Sillevis Smitt P.A.E. (2011). Addition of serum-containing medium to cerebrospinal fluid prevents cellular loss over time. J. Neurol..

[118] Greig B., Stetler-Stevenson M., Lea J. (2014). Stabilization media increases recovery in paucicellular cerebrospinal fluid specimens submitted for flow cytometry testing. Cytom. B Clin. Cytom..

[119] de Jongste A.H., Kraan J., van den Broek P.D., Brooimans R.A., Bromberg J.E., van Montfort K.A., Smitt P.A.S., Gratama J.W. (2014). Use of TransFix™ cerebrospinal fluid storage tubes prevents cellular loss and enhances flow cytometric detection of malignant hematological cells after 18 hours of storage. Cytom. B Clin. Cytom..

[120] Singh G., van Laarhoven A., Adams R., Reid T.D., Combrinck J., van Dorp S., Riou C., Thango N., Enslin J., Kruger S. (2024). The influence of fixation and cryopreservation of cerebrospinal fluid on antigen expression and cell percentages by flow cytometric analysis. Sci. Rep..

[121] Zeman D., Revendova K., Bunganic R., Ryzi M., Masarovicova P., Kusnierova P., Kotrlova V., Hradilek P., Stejskal D., Thon V. (2023). Analysis of cerebrospinal fluid cells by flow cytometry: Comparison to conventional cytology. Biomed. Pap. Med. Fac. Palacky Univ. Olomouc.

[122] Zhang L., Yasumizu Y., Deerhake M.E., Moon J., Buitrago-Pocasangre N., Russo A., Wang H., Zhu B., Seibyl J.P., Reddy V. (2025). Inflamed Microglia like Macrophages in the Central Nervous System of Prodromal Parkinson’s Disease. bioRxiv.

[123] Marsh S.E., Walker A.J., Kamath T., Dissing-Olesen L., Hammond T.R., de Soysa T.Y., Young A.M.H., Murphy S., Abdulraouf A., Nadaf N. (2022). Dissection of artifactual and confounding glial signatures by single-cell sequencing of mouse and human brain. Nat. Neurosci..

[124] Rohlwink U.K., Chow F.C., Wasserman S., Dian S., Lai R.P., Chaidir L., Hamers R.L., Wilkinson R.J., Boulware D.R., Cresswell F.V. (2020). Standardized approaches for clinical sampling and endpoint ascertainment in tuberculous meningitis studies. Wellcome Open Res..

[125] Teunissen C., Petzold A., Bennett J., Berven F., Brundin L., Comabella M., Franciotta D., Frederiksen J., Fleming J., Furlan R. (2009). A consensus protocol for the standardization of cerebrospinal fluid collection and biobanking. Neurology.

[126] Ramos-Vicente D., Monterosso P., de Fàbregues O., Roch G., Vila M., Bové J. (2025). Single-cell transcriptomics for immune profiling of cerebrospinal fluid in neurological diseases. Front. Immunol..

[127] Singh G., Tucker E.W., Rohlwink U.K. (2022). Infection in the developing brain: The role of unique systemic immune vulnerabilities. Front. Neurol..

[128] Kim J., Erice C., Rohlwink U.K., Tucker E.W. (2022). Infections in the Developing Brain: The Role of the Neuro-Immune Axis. Front. Neurol..

[129] Cecchini D., Ambrosioni J., Brezzo C., Corti M., Rybko A., Perez M., Poggi S., Ambroggi M. (2007). Tuberculous meningitis in HIV-infected patients: Drug susceptibility and clinical outcome. AIDS.

[130] Cain D.W., Cidlowski J.A. (2017). Immune regulation by glucocorticoids. Nat. Rev. Immunol..

